# On‐Demand Controlled Release Multi‐Drugs Delivery System for Spatiotemporally Synergizing Antitumor Immunotherapy

**DOI:** 10.1002/advs.202414233

**Published:** 2025-01-10

**Authors:** Chenglin Liang, Hanxiao Yang, Tongtong Li, Xiaojuan Jiang, Xinni Li, Chen Gao, Lin Hou

**Affiliations:** ^1^ School of Pharmaceutical Sciences Key Laboratory of Targeting Therapy and Diagnosis for Critical Diseases Zhengzhou University Zhengzhou 450001 China

**Keywords:** CTLs activation, muti‐drugs delivery, PD‐L1 trap release as needed, spatiotemporally synergizing

## Abstract

Although cytotoxic T lymphocytes (CTLs) activation combined with programmed cell death‐1 (PD‐1)/programmed cell death ligand‐1 (PD‐L1) axis blockade have emerged as an effective strategy to improve immunotherapeutic potency, it remains challenging to realize the spatiotemporal synergy of these two components. Herein, the study reports an engineered bacterial‐based delivery system that can simultaneously promote CTLs infiltration and control PD‐L1 binding protein (PD‐L1 trap) release on demand at tumor site. The drug release button of this tumor targeting system is the specific temperature, which is accomplished by dual‐modified melanin nanoparticles with photothermal conversion capacity on the engineered bacterial. These dual‐modified nanoparticles can form in situ reservoir of heat supplier and antitumor immunity activator once arriving at tumor microenvironment (TME). Importantly, the study establishes the personalized administration regimen according to TME changes, and perform local laser irradiation to trigger PD‐L1 trap production only in TME when infiltrated CTLs reach the highest level. This work provides a flexible platform for optimizing cancer immunotherapy.

## Graphical abstract

EB@MPCM with spatiotemporal synergistic delivery system can effectively activate cGAS‐STING signaling pathway and realize in situ blockade of PD‐1/PD‐L1 axis at the time of maximal CTLs infiltration in tumor microenvironment (TME).

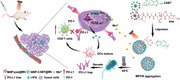

## 1 Introduction

Cytotoxic T lymphocytes (CTLs) are the most powerful effectors in anti‐tumor immunity, and thus many researchers focus on augmenting them to enhance immunotherapy.^[^ ^1^ ^]^ However, programmed cell death ligand‐1 (PD‐L1) on tumor cells that bind with programmed cell death‐1 (PD‐1) results in the dysfunction and depletion of CTLs.^[^ ^2^ ^]^ To address this issue, anti‐PD‐L1 antibody is often combined with immune activators for increasing CTLs infiltration while preventing their depletion.^[^ ^3^ ^]^ But traditional co‐delivery systems are difficult to achieve the controlled blockage of PD‐1/PD‐L1 axis at the exact period when adequate CTLs infiltrate into tumor microenvironment (TME). Although this dilemma can be ameliorated by sequential administration via two individual formulations at pre‐determined time,^[^ ^4^ ^]^ their biodistribution and pharmacokinetics profile is still hard to synergize with each other. Therefore, it is necessary to design a rational delivery system to realize the spatiotemporal synergy of immunity activation and PD‐1/PD‐L1 blockage.

Engineered bacteria technology, which possesses the ability of expressing exogenous protein and preferentially colonizing at tumor site, has been explored in drug delivery system.^[^ ^5^ ^]^ Nevertheless, regulation of the target protein secretion from engineered bacteria according to TME changes is challenging. Design of inducible promoters‐linked genes that are transformed into bacteria provide a promising direction for controlling target protein secretion.^[^ ^6^ ^]^ For example, low pH and hypoxia inducible promoters can achieve spatial control of gene expression in response to acid or hypoxia environment.^[^ ^7^ ^]^ However, these designs cannot personalized trigger target protein production at appropriate time. Consequently, promoters with temporal‐spatial synergy property become the crucial component to adjust the release time of target protein.^[^ ^8^ ^]^ The thermo‐sensitive promoter, which initiates protein expressing process under the specific temperature, is the most potential one. That is, the target protein is only produced and released at set time and region through controlling the local environment temperature. Accordingly, we introduce this kind of promoter into the plasmid containing PD‐L1 binding protein (PD‐L1 trap) sequence and transform *E.coli* BL21, gaining the tumor accumulation system with capacity of controllable PD‐L1 trap release.

In order to reach the temperature triggering PD‐L1 trap secretion and precisely control its release time as well as site, melanin nanoparticles (MNP) with photothermal conversion characteristics offer a perfect option. Fortunately, MNP can adhere on the surface of engineered BL21 (EB) and accumulate at TME through the bacterial hypoxia tendency,^[^ ^9^ ^]^ supplying governable heat to elevate the local temperature under 808 nm laser irradiation on tumor site at determined time. In addition, chemical groups such as phenol hydroxyl and amino groups of MNP can coordinate with metal ions^[^ ^10^ ^]^ and this coordination bond is acid sensitivity.^[^ ^11^ ^]^ Accordingly, Mn^2+^ that is able to activate antitumor immunity^[^ ^12^ ^]^ is chosen to form MNP‐Mn^2+^ complex (MNP@Mn). More importantly, the conceive of coating MNP@Mn on PD‐L1 trap expressed EB lays the foundation for promoting CTLs infiltration and personally regulating PD‐L1 trap release as needed.

Small sized MNP@Mn can be shed from EB due to the low pH and high ROS level at TME,^[^ ^13^ ^]^ but is easily pumped back into the bloodstream, resulting in poor retention at tumor site.^[^ ^14^ ^]^ Consequently, it is tough to maintain the heat source for controlling temperature to produce PD‐L1 trap for a long time. In our previous research, peptide with the sequence of Ala‐Ala‐Asn‐Cys‐Lys (pep) and 2‐cyano‐aminobenzothiazole (CABT) have been used to modify nanomaterials to achieve in situ crosslinking.^[^ ^15^ ^]^ Briefly, pep is spliced by legumain that is abundant in TME to expose 1,2‐thiolamino groups, and reacts with the contiguous cyano groups on CABT through a click cycloaddition reaction. As a result, pep and CABT modification of MNP@Mn (MPCM) will facilitate the small‐sized nanoparticles to crosslink into large aggregates, leading to a prolonged retention in TME.

**Figure 1:**
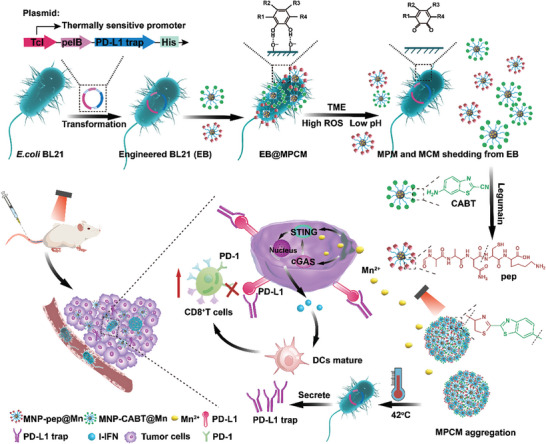
Preparation and anti‐tumor mechanism of EB@MPCM. The plasmid containing the thermosensitive promoter and PD‐L1 trap sequences were transformed into *E.coli* BL21. MNP‐pep@Mn (MPM) and MNP‐CABT@Mn (MCM) adhered to Engineered BL21 (EB) surface. In the tumor microenvironment, MPCM was shed from EB and aggregated under legumain induction. Mn^2+^ can be released under the acid TME and enhance CTLs recruitment as well as infiltration. When the CTLs infiltration rate was the highest, 808 nm laser irradiation was performed to increase the TME temperature and initiated PD‐L1 trap secretion from EB. The PD‐L1 trap bound with PD‐L1 on the tumor cells, blocking the PD‐1/PD‐L1 signaling axis and enhancing CTLs activity.

Herein, we tailor a spatiotemporal synergistic delivery system (EB@MPCM) which can realize in situ blockade of PD‐1/PD‐L1 axis at the time of maximal CTLs infiltration in TME. Once EB@MPCM reaches the tumor site, MPCM is detached from EB and aggregated via legumain induction, forming the long‐time reservoir of Mn^2+^ and heat source. Mn^2+^ can be released under the acid TME and enhance CTLs recruitment as well as infiltration. Notably, 808 nm laser irradiation is performed to increase the TME temperature and initiate PD‐L1 trap secretion from EB, when the CTLs infiltration rate is the highest. In other words, we can accomplish tumor site and time specific PD‐L1 trap release with the change of CTLs in TME so as to optimize T cells‐mediated immunotherapeutic efficacy (**Figure** **1**).

## 2 Results

### 2.1 Synthesis and Characterization of MNP‐pep@Mn (MPM) and MNP‐CABT@Mn (MCM)

**Figure 2:**
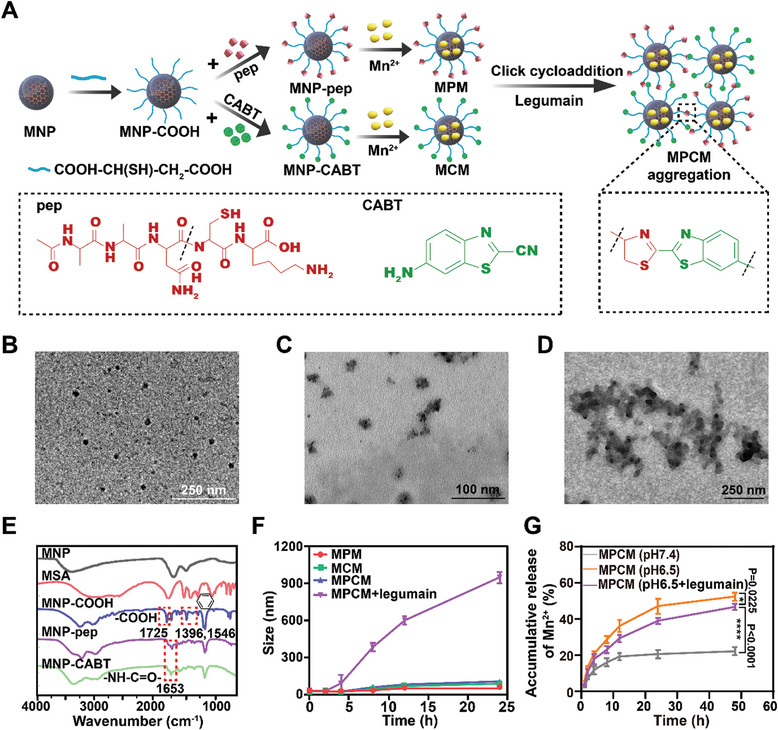
The synthesis and aggregation of MPM and MCM. A) Synthesis rout of MPM and MCM and legumain‐triggered aggregation. B–D) TEM images of MNP (B), MPCM (C), and MPCM incubated with pH 6.5 PBS containing legumain (D). E) FT‐IR spectra of MNP, MSA, MNP‐COOH, MNP‐pep and MNP‐CABT. F) Size change of different preparations within 24 h. G) The release behavior of Mn^2+^ from MPCM under different conditions.

The synthesis procedure of MPM and MCM was shown in **Figure** **2**A. Firstly, melanin nanoparticles (MNP) with average diameter ≈20 nm and zeta potential ≈‐30 mV were obtained by polymerization under alkali conditions. TEM image (Figure 2B) displayed that MNP was homogeneous spheres. Then, MNP was modified with mercaptosuccinic acid (MSA) to yield MNP‐COOH. FT‐IR spectrum (Figure 2E) showed the characteristic peaks of both ‐COOH (1725 cm^−1^) from MSA and benzene (1396 cm^−1^, 1546 cm^−1^) from MNP in MNP‐COOH. ^1^H‐NMR results (Figure S1, Supporting Information) further confirmed the successful synthesis of MNP‐COOH. Next, the legumain responsive polypeptide (Ala‐Ala‐Asn‐Cys‐Lys, pep) and CABT were conjugated with MNP‐COOH by amide reaction to obtain MNP‐pep and MNP‐CABT, respectively. The characteristic peaks of ‐NH‐CO‐ (1653 cm^−1^) appeared in FT‐IR results of MNP‐pep and MNP‐CABT (Figure 2E). ^1^H‐NMR results (Figure S2, Supporting Information) also demonstrated the successful modification of pep and CABT. Finally, Mn^2+^ was loaded on the surface of MNP‐pep and MNP‐CABT to form MNP‐pep@Mn (MPM) and MNP‐CABT@Mn (MCM), respectively, due to the polyphenolic structure of MNP. The complexation efficiency of Mn^2+^ detected by ICP‐MS was ≈3.5%.

Since free ‐NH_2_ and ‐SH on cysteine (Cys) were exposed after pep responded to legumain, which in turn induced MPM cross‐linking with ‐CN on CABT via click‐cycloaddition reaction. The aggregation properties of MPM and MCM in TME were further investigated. As shown in TEM image (Figure 2C), MPM and MCM mixture (MPCM) exhibited uniform dispersion with particle size of ≈20 nm. In comparison, MPCM aggregated obviously after incubation in pH 6.5 PBS containing legumain (simulating TME) (Figure 2D), and the particle size increased from ≈20 nm to ≈1000 nm with incubation time extended (Figure 2F). By contrast, the particle size of MPCM under condition without legumain remained unchanged. Furthermore, the release behavior of Mn^2+^ from MPCM under different conditions was studied (Figure 2G). Approximately 47% and 53% of Mn^2+^ was released from MPCM in pH 6.5 PBS with or without legumain, respectively, while only ≈20% of Mn^2+^ could be released at pH 7.4 PBS after 48 h. This result indicated that Mn^2+^ release from MPCM was dependent on the pH value and rare leakage in the blood circulation, and legumain induced MPCM aggregation slowed down the Mn^2+^ release. These results indicated that Mn^2+^ reservoirs could be formed due to MPCM aggregation and release enough Mn^2+^ in TME, paving the way for cGAS‐STING activation.

### 2.2 Design and Characterization of EB@MPCM

**Figure 3:**
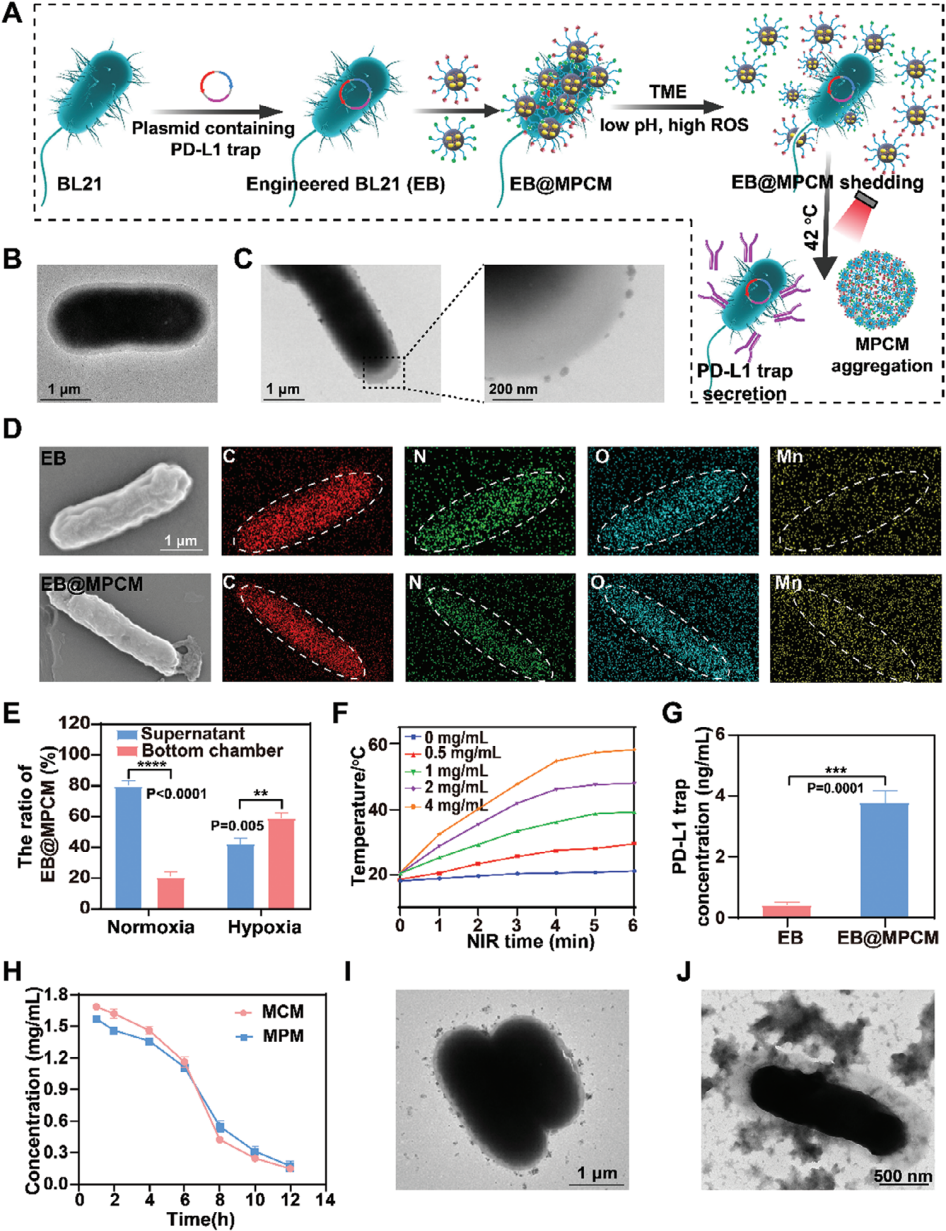
The design and characterization of EB@MPCM. A) The design and synthesis route of EB@MPCM. B,C) TEM images of EB (B) and EB@MPCM (C). D) Elemental mapping of EB and EB@MPCM. E) Flow cytometry analysis of the ratio of EGFP‐labeled EB@MPCM in supernatant and bottom chamber. F) Photothermal curves of different concentration EB@MPCM under 808 nm laser irradiation. G) The expression of PD‐L1 trap in EB and EB@MPCM under 808 nm laser irradiation. H) Concentration changes curves of MCM and MPM in EB@MPCM at different incubation time. I,J) TEM images of EB@MPCM after incubation in PBS (pH 6.8) containing 100 μΜ H_2_O_2_ for 12 h (I) and incubation with legumain (J).

Tumor targeting delivery of MPCM is the basis for generating in situ reservoir of Mn^2+^ and heat source, which trigger the antitumor immunity and local release of PD‐L1 trap. Accordingly, *E.coli* BL21 that possesses the capacity of tumor accumulation and PD‐L1 trap expression was utilized to load MPCM. The design and preparation of EB@MPCM (EB, Engineered BL21 expressing PD‐L1 trap) was described in the **Figure** **3**A, of which the transfected plasmid contained the thermosensitive promoter and PD‐L1 trap sequences. SDS‐PAGE and western blotting results demonstrated that there was an obvious PD‐L1 trap band at 33 kDa in EB group after incubation at 42 °C (Figure S3A,B, Supporting Information), but almost no bands appeared under the condition at 37 °C. This phenomenon confirmed the temperature‐regulated secretion ability of EB. To further determine the secreted amount of PD‐L1 trap, supernatant proteins at different incubation temperature and different time was detected by ELISA kit. As shown in Figure S3C (Supporting Information), compared to at 37 °C, the level of PD‐L1 trap increased significantly after EB incubation at 42 °C for 10 min, and ≈1.3 times higher than that after 5 min of incubation, further verifying the hot‐triggered production of PD‐L1 trap. TEM image displayed that the surface of EB was smooth (Figure 3B), and MPCM could be adhered to EB to form EB@MPCM (Figure 3C). After EB@MPCM was incubated in pH 7.4 PBS (mimics normal physiological environment) for 12 h, MPCM was still existed on the surface of EB, suggesting that MPCM could stably adhere to the surface of EB (Figure S3D, Supporting Information). Elemental mapping result exhibited that more manganese element exists in EB@MPCM than EB (Figure 3D). In addition, MPCM loading had no effect on the EB activity (Figure S3E, Supporting Information). Furthermore, ≈ 51% and ≈ 54% of Mn^2+^ was released from EB@MPCM in pH 6.5 PBS with or without legumain, which were similar to the release profile of MPCM, suggesting MPCM adhesion to EB did not affect Mn^2+^ release (Figure S3F, Supporting Information).

The hypoxic tendency mediated tumor targetability of EB@MPCM was examined in simulated hypoxic TME, and EB was labeled by enhanced green fluorescent protein (EGFP). Flow cytometry and fluorescence microscopy analysis (Figure 3E; Figure S3G, Supporting Information) showed that more EB@MCPM appeared in the hypoxia environment, compared with that in normoxia environment. With the incubation time extended, the amount of EB@MCPM in hypoxic environment increased, and was about five times more than that in normal oxygen condition at 45 min (Figure S3H, Supporting Information).

Since the secretion of PD‐L1 trap is initiated at 42 °C, photothermal conversion property of EB@MPCM is crucial during this process. As demonstrated in Figure S3I (Supporting Information), the temperature of MNP‐based preparations, including MPM, MCM, MPCM, and EB@MPCM, rose continuously with the extension of time under 808 nm laser irradiation and reached 42 °C within 3 min. By contrast, EB showed negligible temperature change under the same conditions due to the lack of photothermal agent. Figure 3F and Figure S3J (Supporting Information) presented that 2 mg/mL of EB@MPCM reached 42 °C within 3 min under 808 nm laser irradiation. And the concentration of PD‐L1 trap secreted by EB@MPCM was about tenfold higher than that in EB group after irradiation (Figure 3G). Moreover, OD600 value of bacterial medium suggested that MPCM modification did not affect EB growth even under 808 nm laser irradiation (Figure S3K, Supporting Information). In addition, the concentration of MPM and MCM on EB decreased gradually in pH 6.8 PBS containing H_2_O_2_ (simulating TME), indicating that the nanoparticles were shed from EB@MPCM (Figure 3H). The reason is that the hydrogen bonds between MPCM and EB are broken due to the high ROS level and low pH in the TME, and the phenolic hydroxyl groups of MPM and MCM are oxidized and protonated to form quinones, resulting in their detachment from EB.^[^ ^16^ ^]^ As exhibited in Figure 3I,J, scattered nanoparticles were located around EB after EB@MPCM was treated in pH 6.8 PBS containing H_2_O_2_ for 12 h, and above nanoparticles became aggregation, accompanied by individual bacterial appeared when legumain was added into the medium. Taken together, EB@MPCM possessed the potential of tumor accumulation, and could self‐assemble into hot reservoir and in situ PD‐L1 trap generator, facilitating the therapeutic outcomes.

### 2.3 In Vitro Evaluation of Antitumor Immunity Related Pathway

In the previous research, we have verified that Mn^2+^ can activate the cGAS‐STING pathway of tumor cells, thus improving the adaptive immune response. Herein, the expression of cGAS‐STING pathway‐related protein (including pTBK1 and pIRF3) were further examined in 4T1 tumor cells by western blotting, and demonstrated a Mn^2+^ concentration dependent manner (Figure S4A, Supporting Information). Considering the cytotoxicity of Mn^2+^, we chose 0.4 mm of Mn^2+^ for activating the cGAS‐STING pathway. Furthermore, compared with MNP and MNP+Laser groups, MPCM based groups significantly up‐regulated the expression of pTBK1 and pIRF3. Moreover, the pathway activation was not affected by laser irradiation (Figure S4B, Supporting Information).

Reportedly, cGAS‐STING pathway activation upregulates PD‐L1 level.^[^ ^17^ ^]^ Therefore, the influence of cGAS‐STING pathway on PD‐L1 expression was evaluated in 4T1 tumor cells by flow cytometry (Figure S4C, Supporting Information). It can be seen that MPCM upregulated the expression of PD‐L1 on 4T1 tumor cells, PD‐L1 trap blocked their binding with PD‐L1 antibody and resulted in the decreased detection amount. Consistent with this phenomenon, PD‐L1 level remained low after co‐incubation of MPCM and PD‐L1 trap with 4T1 tumor cells. These results indicated that MPCM effectively activated cGAS‐STING signaling pathway but up‐regulated the expression of PD‐L1 on the tumor cells. Fortunately, this elevated PD‐L1 expression can be blocked by PD‐L1 trap, thus improving the CTLs function (Figure S4D, Supporting Information).

### 2.4 In Vivo Distribution of EB@MPCM

**Figure 4:**
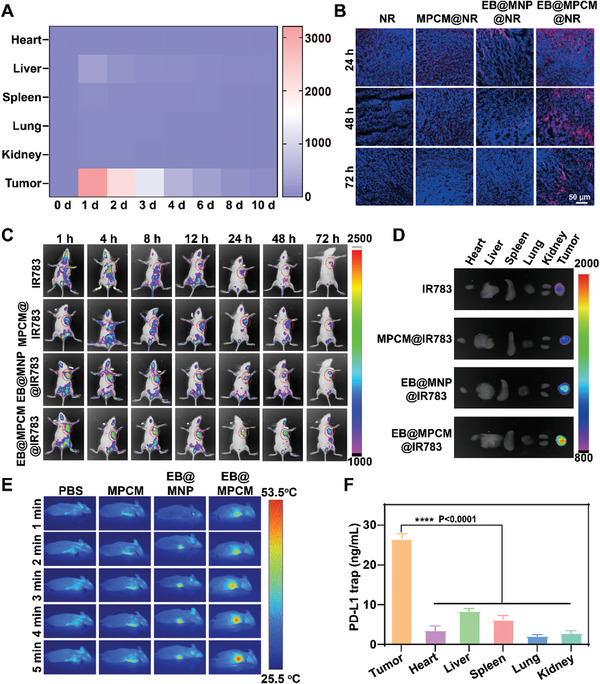
In vivo distribution of EB@MPCM and PD‐L1 trap release induced by photothermal in 4T1 tumor‐bearing mice. A) Heat map of EB@MPCM distribution in different tissues at different times. B) In vivo retention of free NR, MPCM@NR, EB@MNP@NR, and EB@MPCM@NR in tumor site at 24, 48, and 72 h after intravenous injection. Blue: cell nucleus; Red: preparations labeled with nile red (NR). C) In vivo imaging of 4T1 tumor‐bearing mice at different time points after intravenous injection of different formulations. The red circles represent the tumor site. D) Ex vivo imaging of different formulations in major organs at 72 h post‐injection. E) Representative infrared images of tumor tissues of 4T1 tumor‐bearing mice treated with PBS, MPCM, EB@MNP, and EB@MPCM under 808 nm laser irradiation. F) Expression of PD‐L1 trap in different tissues of 4T1 tumor‐bearing mice after EB@MPCM treatment under 808 nm laser irradiation.

Effective accumulation of EB@MPCM at tumor site is critical for achieving desirable anti‐tumor immunotherapy. In view of this, the biodistribution of EB@MPCM was studied by counting bacterial colonies in different tissues and in vivo fluorescence imaging. As shown in **Figure** **4**A and Figure S5 (Supporting Information), the number of EB in the heart, lung and kidney was negligible after administration. By contrast, a substantial quantity of bacterial colonies was observed in the tumor tissues, which were ≈14 times and ≈49 times higher than those in liver and spleen, respectively, owing to the hypoxic tendency of EB. Furthermore, in vivo imaging (Figure 4C; Figure S6A, Supporting Information) exhibited the consistent results that IR783 labeled EB@MPCM (EB@MPCM@IR783) presented the strongest fluorescent signal at tumor site, and the fluorescence signal was similar at 24 h and 48 h. Although EB@MNP@IR783 also showed an obvious signal at tumor site, the intensity became weaker with the extension of time, and was significantly lower than that of EB@MPCM@IR783. It was because the small‐sized MNP tended to backflow into bloodstream, resulting in the poor retention. While the aggregation of MPCM induced by legumain in TME prevented this phenomenon, resulting in a prolonged retention at the tumor site. Ex vivo fluorescence imaging (Figure 4D; Figure S6B, Supporting Information) also demonstrated the superior tumor targeting and retention effect of EB@MPCM. To further confirm that EB@MPCM can accumulate at the tumor site, the Mn content in tumor tissues of MPCM and EB@MPCM treated 4T1 tumor‐bearing mice was measured by ICP‐MS. As shown in Figure S6D (Supporting Information), although the Mn content in tumor tissues of MPCM and EB@MPCM groups was low for 1 h, the Mn content in EB@MPCM group was significantly higher than that in MPCM group with the extension of time. Furthermore, the Mn content in tumor tissues for EB@MPCM reached a maximum value (≈ 4.9%) at 8 h, and even remained ≈ 4.7% at 24 h, which were also higher than that in MPCM group. It suggested that MPCM did not shed from the EB after system delivery, and arrived at the tumor microenvironment due to the hypoxic tendency of EB. In addition, tumor tissue slices of 4T1 tumor‐bearing mice treated with nile red (NR)‐labeled EB@MPCM were analyzed (Figure 4B; Figure S6C, Supporting Information). Compared with free NR, MPCM@NR and EB@MNP@NR, EB@MPCM@NR presented intense and large area of red fluorescence even at 72 h, further confirming its prolonged retention capacity.

In vivo photothermal efficiency of EB@MPCM were investigated by monitoring the temperature at tumor site under 808 nm laser irradiation (Figure 4E). We found that the temperature in EB@MPCM group rapidly increased to 42 °C within 3 min, which was much higher than that in PBS, MPCM and EB@MNP groups. Correspondingly, the concentration of PD‐L1 trap secreted at tumor site in EB@MPCM group was remarkably more than that in other tissues (Figure 4F). These results suggested that EB@MPCM possessed excellent tumor targeting and enhanced retention effect, thus generating a heat source at tumor site and triggering in situ secretion of PD‐L1 trap.

### 2.5 Antitumor Efficacy of EB@MPCM in 4T1 Tumor‐Bearing Mice

Encouraged by above results, the anti‐tumor effect of EB@MPCM in 4T1 tumor‐bearing mice was investigated. To achieve ideal therapeutic potency, administration regimen was determined by detecting the expression levels of CD8^+^T cells and PD‐L1^+^ tumor cells in tumor tissues using flow cytometry. As shown in the Figure S7A (Supporting Information), the number of infiltrating CD8^+^T cells was maximal on day 3 after EB@MPCM treatment (increased from 5.73% to 22.0%), and downregulated to 15.9% on day 4. In addition, the expression of PD‐L1 on the tumor cells also elevated from 9.44% to 22.8% on day 3 (Figure S7B, Supporting Information). As a result, 808 nm laser irradiation could be performed on day 3 to induce PD‐L1 trap production for optimal blockade of PD‐L1 on tumor cells.

**Figure 5:**
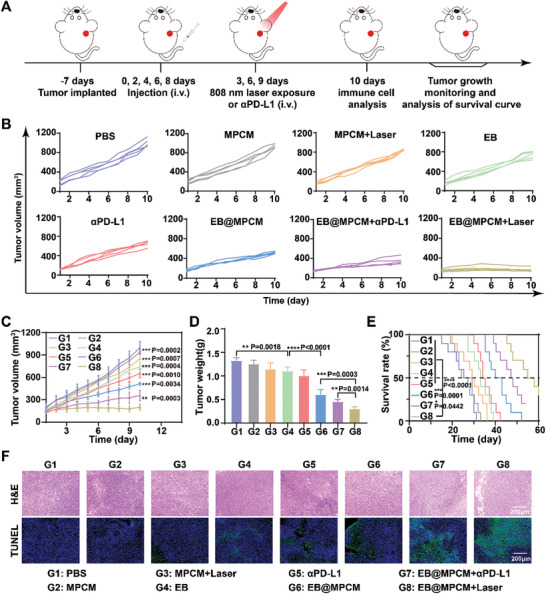
Antitumor efficacy of EB@MPCM in 4T1 tumor‐bearing mice. A) Treatment schedule on 4T1 tumor‐bearing mice. B,C) Individual tumor growth curve (B) and average tumor growth curve (C) during treatment. D,E) Tumor weight (D) and survival rate (E) of 4T1 tumor‐bearing mice treated with different preparations. F) Representative images of H&E and TUNEL staining of tumor slices.

Subsequently, the anti‐tumor effect of EB@MPCM was evaluated according to the regimen (**Figure** **5**A). When the tumor volume reached ≈100 mm^3^, 4T1 tumor‐bearing mice were randomly divided into 10 groups, including PBS, PBS+Laser, MPCM, MPCM+Laser, EB, EB+Laser, αPD‐L1, EB@MPCM, EB@MPCM+ αPD‐L1 and EB@MPCM+Laser. The tumor volume and body weight were recorded every day. As exhibited in Figure S8B,C (Supporting Information), compared to PBS, PBS+Laser and EB+Laser hardly suppressed tumor growth, signifying that laser and EB didn't possess antitumor effects. In addition, the tumor growth was also hardly inhibited in MPCM and MPCM+Laser group, owing to the non‐tumor targeting disadvantage of MPCM (Figure 5B–D). EB and αPD‐L1 showed weak tumor inhibition of ≈17% and ≈24%, respectively. In comparison, the tumor inhibition rate in EB@MPCM group reached ≈54%, because it could accumulate and retain at the tumor site. Notably, after combination with αPD‐L1, EB@MPCM significantly repressed tumor growth with an inhibition rate of ≈66%, which was still weaker than that of the EB@MPCM+Laser group (≈77%). It was attributed to in situ release of PD‐L1 trap under 808 nm laser irradiation, thus boosting the cytotoxicity of T cells. Ex vivo tumor tissues images also confirmed the results (Figures S8A and S9A, Supporting Information). Moreover, hematoxylin and eosin (H&E) and terminal deoxynucleotidyl transferase‐mediated deoxy uridine triphosphate nick end labeling (TUNEL) staining of tumor tissues results showed the most serious damage and widespread apoptosis in EB@MPCM+Laser group (Figure 5F).

To further verify the superior tumor inhibition ability of EB@MPCM+Laser, the survival rate of 4T1 tumor‐bearing mice treated with different formulations was assessed. In Figure 5E, EB@MPCM+Laser group displayed the highest median survival time with 55 days, compared with PBS (25 days), MPCM (24 days), MPCM+Laser (29 days), EB (33 days), αPD‐L1 (34 days), EB@MPCM (40 days) and EB@MPCM+αPD‐L1 (47 days).

After that, the biosafety was analyzed. There was no significantly decrease in body weight in all treatment groups (Figures S8D and S9B, Supporting Information). H&E staining of the heart, liver, spleen, lungs and kidneys showed no obvious damage (Figure S9C, Supporting Information), suggesting that EB@MPCM possessed good biocompatibility and biosafety.

### 2.6 Anti‐Tumor Mechanism of EB@MPCM in 4T1 Tumor‐Bearing Mice

**Figure 6:**
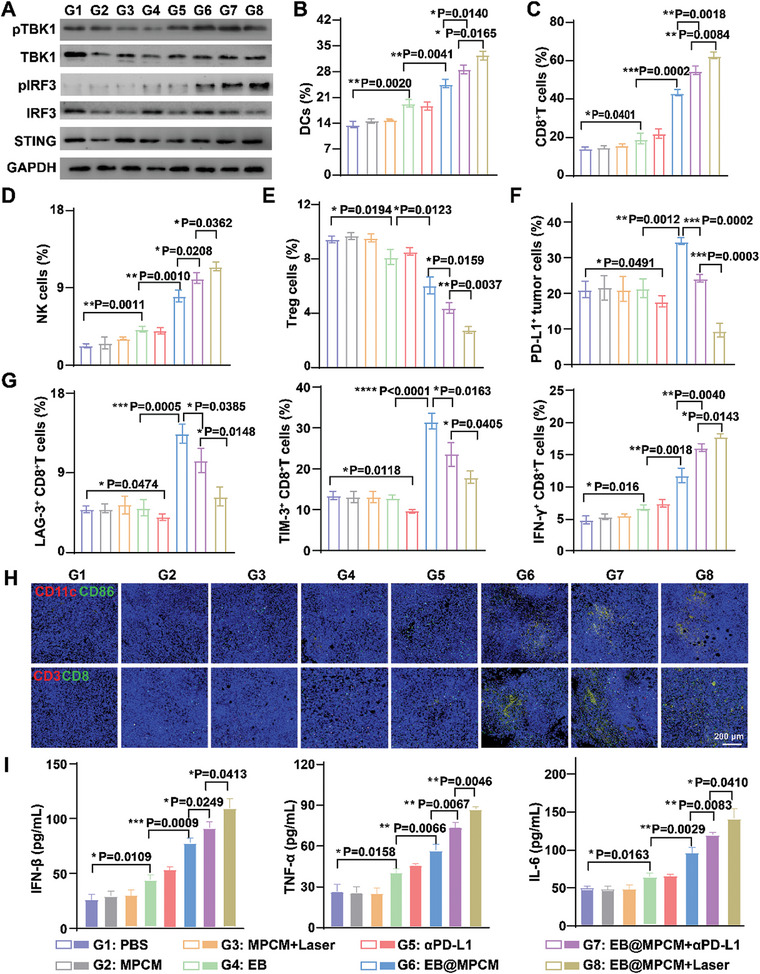
Anti‐tumor mechanism of EB@MPCM in 4T1 tumor‐bearing mice. A) Expression of cGAS‐STING signaling pathway‐related protein in tumor tissues after treatment with different preparations. B–G) Semiquantitative analysis of DCs (B), CD8^+^T cells (C), NK cells (D), Treg cells (E), PD‐L1^+^ tumor cells (F), LAG‐3^+^CD8^+^T cells, TIM‐3^+^CD8^+^T cells and IFN‐γ^+^CD8^+^T cells (G) in tumor tissues after treatment with different preparations. H) Representative immunofluorescence images of DCs and CD8^+^T cells infiltration in tumor tissues. I) Expression of IFN‐β, TNF‐α, and IL‐6 in tumor tissues after treatment.

To explore anti‐tumor mechanism, we evaluated TME changes after EB@MPCM+Laser treatment, including cGAS‐STING pathway related proteins, immune cells and cytokines. As shown in **Figure** **6**A and Figure S10A (Supporting Information), the expression of pTBK1 and pIRF3 in tumor tissues was dramatically upregulated in EB@MPCM based groups, because EB facilitated MPCM to accumulate at tumor site and form Mn^2+^ reservoir, leading to effective activation of cGAS‐STING pathway. Subsequently, type I IFN was promoted to release, thereby stimulating DC cells maturation, cytotoxic T cells infiltration, and NK cells recruitment.^[^ ^18^ ^]^ However, cGAS‐STING pathway activation induces PD‐L1 upregulation on tumor cells, resulting in the CD8^+^T cells depletion. Accordingly, immune cells changes and CD8^+^T cells function were analyzed by flow cytometry. In Figure 6B and Figure S11A (Supporting Information), EB@MPCM increased the proportion of mature DCs (from 13.5% to 23.6%) due to the activation of cGAS‐STING pathway. However, it was weaker than that in EB@MPCM+αPD‐L1 (29.2%), possibly because PD‐1/PD‐L1 blockade could facilitate immune effector cells, such as CD8^+^T cells and NK cells, to secrete more cytokines (TNF‐α, IFN‐γ, IL‐6, etc.), which in turn promoted DCs maturation. For EB@MPCM+Laser group, in situ release of PD‐L1 trap at tumor site enhanced this effect (35.2% of DC maturation), and the proportion of CD8^+^T cells reached the highest level (60.5%) (Figure 6C; Figure S11B, Supporting Information). Consistently, immunofluorescence staining results (Figure 6H; Figure S10B,C, Supporting Information) displayed an increased infiltration of DCs and CD8^+^T cells in tumor tissues after EB@MPCM+Laser treatment.

PD‐L1 trap secreted by EB at the suitable time possessed the potential to improve CD8^+^T cells activity. As exhibited in Figure 6F and Figure S12A (Supporting Information), PD‐L1^+^ tumor cells in TME elevated from ≈18.5% to 34.6% after EB@MPCM therapy. After combination with αPD‐L1, PD‐L1 level was reduced to 23.3%. More significantly, PD‐L1 level was only 16.6% in EB@MPCM+Laser group, and the inhibitory molecules (TIM‐3 and LAG‐3) were also decreased to ≈16.9% and ≈5.1%, respectively. Furthermore, EB@MPCM+Laser showed the highest abundance of IFN‐γ, which is the main effector molecule for CD8^+^T cells to exert killing function (Figure 6G; Figure S12B–D, Supporting Information).

In addition, EB@MPCM+Laser triggered ≈11.1% of NK cells infiltration within TME (Figure 6D; Figure S11C, Supporting Information). Notably, the reduction in regulatory T cells (Tregs) (Figure 6E; Figure S11D, Supporting Information) and myeloid‐derived suppressor cells (MDSCs) (Figures S10D and S11E, Supporting Information) was observed in TME after EB@MPCM+Laser treatment. Inflammatory factors, such as IFN‐β, IL‐6, TNF‐α, and IFN‐γ, were also dramatically augmented (Figure 6I; Figure S10E, Supporting Information). Above results implied that EB@MPCM+Laser increased effector immune cells infiltration, strengthen CD8^+^T cells killing efficacy, and improved immunosuppressive TME, leading to an improved anti‐tumor immune response.

### 2.7 Anti‐Tumor and Immunomodulatory Effects on B16‐F10 Tumor‐Bearing Mice

**Figure 7:**
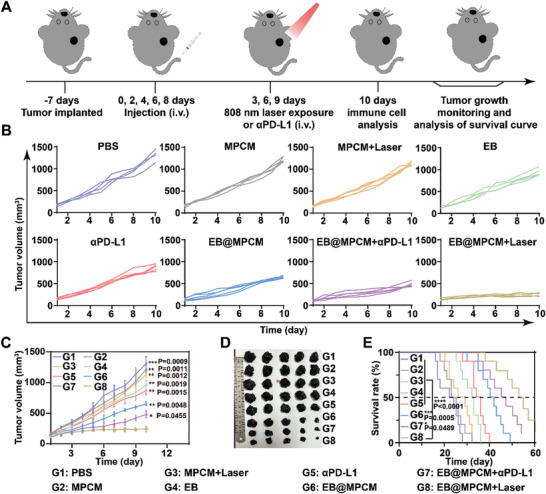
Antitumor efficacy of EB@MPCM in B16‐F10 tumor‐bearing mice. A) Treatment schedule on B16‐F10 tumor‐bearing mice. B,C) Individual tumor growth curve (B) and average tumor growth curve (C) during treatment. D,E) Ex vivo tumor images (D) and survival rate (E) of B16‐F10 tumor‐bearing mice treated with different preparations.

To further evaluate the applicability of EB@MPCM+Laser in anti‐tumor immunotherapy, B16‐F10 tumor‐bearing mice were chosen as the second model (**Figure** **7**A). As shown in Figure 7B–D and Figure S13A (Supporting Information), EB@MPCM+Laser treatment demonstrated the strongest anti‐tumor efficacy, with ≈80% of inhibition rate. This superior effect stemmed from the formation of Mn^2+^ reservoirs and thermoresponsive release of PD‐L1 trap in TME, leading to the improved anti‐tumor immunity. In addition, all treatments did not induce significant changes in body weight (Figure S13B, Supporting Information), indicating good biosafety. Furthermore, EB@MPCM+Laser treatment extended the survival of B16‐F10 tumor‐bearing mice (from 22 to 53 days) (Figure 7E). That is, EB@MPCM+Laser has the same excellent anti‐tumor effect on B16‐F10 tumor‐bearing mice.

Then, the changes of immune cells in TME after EB@MPCM+Laser treatment were investigated. It revealed that the proportion of mature DC cells in EB@MPCM+Laser group was as high as ≈43.2% (Figure S14A, Supporting Information), while CD8^+^T cells number elevated to ≈46.7% (Figure S14B, Supporting Information) and infiltration rate of NK cells increased from ≈2.2% to ≈11.7% in TME (Figure S14C, Supporting Information). Immunosuppressive cells, such as Treg cells (Figure S14D, Supporting Information) and MDSCs (Figure S14E, Supporting Information), displayed a reduction after EB@MPCM+Laser treatment. As mentioned above, cGAS‐STING pathway activation induced PD‐L1 upregulation, in turn affecting the activity of CD8^+^T cells. Accordingly, PD‐L1 expression level and CD8^+^T cells activity in B16‐F10 tumor tissues was detected by flow cytometry. In Figure S15A (Supporting Information), compared with PBS, PD‐L1 level on tumor cells increased by ≈6.7% after EB@MPCM therapy, but was down‐regulated after EB@MPCM+αPD‐L1 or EB@MPCM+Laser treatment, especially in EB@MPCM+Laser group. Meanwhile, LAG‐3 (Figure S15B, Supporting Information) and TIM‐3 (Figure S15C, Supporting Information) on CD8^+^T cells in EB@MPCM+Laser group were lower than those in EB@MPCM+αPD‐L1 group by ≈13.6% and ≈7%, respectively. As expected, EB@MPCM+Laser evoked the highest proportion of IFN‐γ^+^CD8^+^T cells in tumor tissues (Figure S15D, Supporting Information). These results suggested that EB@MPCM+Laser has perfect anti‐tumor efficacy in both cold tumor (4T1 tumor model) and hot tumor (B16‐F10 tumor model).

## 3 Discussion

Combination of increasing CTLs infiltration with anti‐PD‐L1 therapy has become an encouraging strategy to enhance antitumor effects. Notably, blocking PD‐1/PD‐L1 axis at the exact time when CTLs amount reach the highest in TME is probably to maximize the antitumor immunity. However, coordinating the biodistribution and pharmacokinetics behavior of different active ingredients to achieve their spatiotemporal synergism remains challenging.

To this end, we designed a thermal‐promoting PD‐L1 trap secretion plasmid and took advantages of the engineered bacteria technology to construct on‐demand delivery system (EB@MPCM). In this system, MPCM with laser photo‐thermal conversion capacity was adhered on EB to provide the heat for reaching the temperature that triggered PD‐L1 trap production. Detailly, EB@MPCM with hypoxic tendency preferentially accumulated at tumor site, and MPCM shedding from EB formed aggregates with particle size ≈1000 nm by legumain induction. Our experimental data demonstrated that the retention time of MPCM aggregates in TME was up to 72 h, which could act as a Mn^2+^ reservoir and heat source. The released Mn^2+^ effectively activated cGAS‐STING pathway, augmenting CTLs recruitment and infiltration. By monitoring the TME changes at different time, we found the CTLs infiltration rate and PD‐L1 expression on tumor cells reached the highest level on the 3rd day after administration. Accordingly, 808 nm laser irradiation was performed on tumor site at this time to adjust the temperature and trigger in situ PD‐L1 trap secretion, ultimately accomplishing the strongest enhancement of CTLs activity and optimal blockade of PD‐1/PD‐L1 axis.

In conclusion, EB@MPCM with spatiotemporal synergistic effect effectively activated cGAS‐STING signaling pathway and targeted blockade of PD‐1/PD‐L1 axis on demand, thereby provoking robust anti‐tumor immune response and significantly inhibiting tumor growth as well as prolonging survival in both cold tumor model and hot tumor model. Our work provided a new direction for amplifying CTLs‐mediated tumor immunotherapy.

## Conflict of Interest

The authors declare no conflict of interest.

## Author Contributions

L.H. conceived the project and designed the research. L.H. and C.L. wrote and revised the manuscript. C.L., H.Y., and T.L. performed the experiments and data analysis. X.J., X.L., and C.G. assisted in completing the experiments.

## Supporting information



Supporting Information

## Data Availability

The data that support the findings of this study are available from the corresponding author upon reasonable request.
