# Supplementary material for: On‐Demand Controlled Release Multi‐Drugs Delivery System for Spatiotemporally Synergizing Antitumor Immunotherapy
Source: Adv Sci (Weinh). 2025 Jan 10;12(9):2414233. doi: 10.1002/advs.202414233 (PMC11884579; doi:10.1002/advs.202414233)
Supplement: Supplementary file 1 — Supporting Information [file ADVS-12-2414233-s001.docx]

Supplementary Information

**On-demand controlled release multi-drugs delivery system for spatiotemporally synergizing antitumor immunotherapy**

*Chenglin Liang^1^, Hanxiao Yang^1^, Tongtong Li^1^, Xiaojuan Jiang^1^, Xinni Li^1^, Chen Gao^1^, Lin Hou^1^**

*^1^School of Pharmaceutical Sciences, Key Laboratory of Targeting Therapy and Diagnosis for Critical Diseases, Zhengzhou University, Zhengzhou 450001, China*

**Corresponding Author: Email address: houlin@zzu.edu.cn (Lin Hou)*

**Materials and Methods**

**Materials and characterizations**

Melanin was purchased from Sigma–Aldrich. Sodium hydroxide, hydrochloric acid (concentration>37%) and phosphate buffer (PBS, pH 7.4) was purchased from Tianjin Fengchuan Chemical Reagent Technology Co., Ltd (Tianjin, China). Mercaptosuccinic acid, 1-(3-Dimethylaminopropyl)-3-ethylcarbodiimide hydrochloride (EDC·HCl) and N-hydroxy succinimide (NHS) were purchased from Shanghai Aladdin Biochemical Technology Co., Ltd. (Shanghai, China). 2-Amino-benzothiazole-6-carbonitrile (CABT) was purchased from Shanghai Superb Chemical Technology Co., Ltd. (Shanghai, China). Polypeptide chain (Ala-Ala-Asn-Cys-Lys, concentration>95%) was purchased from Zhengzhou Paihe Tede Pharmaceutical Technology Co., Ltd. (Zhengzhou, China). Legumain was purchased from Suzhou Nearshore Protein Technology Co., Ltd. pBV220-pelB-PD-L1 trap was purchased from Wuhan Miaoling Biotechnology Co., Ltd. Tris (hydroxymethyl) aminomethane was purchased from Beijing Solaibao Technology Co., Ltd. Manganese chloride tetrahydrate was purchased from Shanghai Macklin Biochemical Technology Co., Ltd. Trichloroacetic acid kit was purchased from Jiangsu Kaiji Biotechnology Co., Ltd. His tag ELISA detection kit was purchased from GenScript Biotech Co., Ltd. p-TBK1 (Cat# 5493), p-IRF3 (Cat# 4947), TBK1 (Cat# 3503), IRF3 (Cat# 4302), STING (Cat# 13647) antibodies were purchased from Cell Signaling Technology.

Morphology observed through TEM (Talos L120C, Czech Republic) and SEM (Helios G4 CX, Czech Republic). Size and zeta potential were measured by DLS (Zetasizer Nano-ZS90, UK). FT-IR spectra was measured by infrared spectrometer (Nicolet iS10, USA). OD value was obtained by BioTek Synergy H1 (H1MF, USA). Immune cells detection was analyzed by flow cytometry (BD Accuri C6, USA). Infrared thermography is captured by an infrared thermography instrument (Ti-200, USA). The distribution images of different formulation groups in mice and *in vitro* tissues were captured by IVIS (FX PRO, USA).

**Cell lines and Animals**

The murine breast cancer cell lines (4T1) were obtained from Wuhan Prosser Life Technology Co., Ltd. and cultured in Roswell Park Memorial Institute (RPMI) 1640 (Solarbio) medium with 10% fetal bovine serum. BALB/c mice and C57BL/6J (6-8 weeks old, female, 18-20 g) were purchased from SPF Biotechnology Co., Ltd. (Beijing, China). All animal procedures were performed in accordance with the Guidelines for Care and Use of Laboratory Animals of Zhengzhou University (# yxy11sc20230093).

**Preparation of MNP**

Melanin (10 mg) was dissolved in 0.1 M NaOH solution under vigorous stirring. After dissolution, the pH was adjusted to 7.0 with HCl under ultrasound. Then, the solution was dialyzed in a dialysis bag (MWCO: 12000 Da) for 48 h and freeze-dried to obtain melanin nanoparticles (MNP).

**Preparation of MNP-COOH**

Mercaptosuccinic acid (6 mg) was added to 10 mL of Tris buffer (pH 8.5) containing MNP (1 mg/mL). The solution was stirred at room temperature for 6 h and dialyzed for 48 h to remove unreacted MSA. MNP-COOH was obtained after freeze-drying.

**Preparation of MPM and MCM**

EDC (10 mg) and NHS (6 mg) were added to MNP-COOH solution (10 mg/mL), respectively. The solution was stirred at room temperature for 3 h to activate the carboxyl group. Then, 7 mg of pep was added to the above solution. After adjusting the pH to 8.0, the solution was stirred at room temperature under nitrogen protection for 24 h and dialyzed to obtain MNP-pep. MNP-CABT was synthesized by the same method.

For the preparation of MPM and MCM, 10 mg of MNP-pep or MNP-CABT and 1 mg of MnCl_2_·4H_2_O were dissolved in PBS, respectively. The mixture was stirred at 40℃ for 1 h. MPM or MCM was obtained by dialyzing and freeze-drying. The content of Mn^2+^ was detected by ICP-MS.

**Preparation of EB@MPCM**

The engineered *E. coli* BL21 containing the PD-L1 trap-6×his was purchased from Wuhan Miaoling Bioscience & Technology Co. Ltd. The gene sequences of pelB and PD-L1 trap were shown in Table S1. MPM and MCM were dispersed in PBS and 1×10^8^ CFU engineered *E. coli* BL21 was added into the above solution. Then the solution was stirred at room temperature for 3 h. EB@MPCM was collected by centrifugation (4℃, 5000 rpm, 10min), and washed three times with PBS.

**Aggregation of MPCM**

MPM, MCM and MPCM were dispersed in pH 6.5 PBS with or without legumain (2.5 μg/mL) and incubated in a shaking incubator (37℃, 150 rpm) for different times. The particle size of samples was measured by DLS and the morphology of samples was observed by TEM.

**The release behavior of Mn^2+^ from MPCM and EB@MPCM under different conditions**

The dialysis bag method was used to determine the Mn^2+^ release profiles. 1 mL of MPCM and EB@MPCM was added to dialysis bag, which was then placed into a tube with 20 mL of pH 7.4 PBS or pH 6.8 PBS containing legumain. The tubes were placed in shaker at 37℃ (100 rpm/min). 1 mL dialysis medium was collected at predetermined time points and replenished with the corresponding volume. The concentration of released Mn^2+^ was detected by ICP-MS.

**The expression of PD-L1 trap**

EB was incubated in shaker (200 rpm, 10 min) at 37℃ and 42℃, respectively. The EB was centrifuged at 12000 rpm/min for 10 min at 4℃, and supernatant was collected. Proteins in supernatant were obtained by concentrating protein with trichloroacetic acid kit (KeyGEN BioTECH, KGB1101-100). Then, protein samples were separated with SDS polyacrylamide gel electrophoresis and the gel was captured by ImageLab system (Bio-Rad).

For western blotting analysis of PD-L1 trap, the gel was transferred to PVDF membrane. After blocking with 5% nonfat milk for 1 h, the membrane was incubated with PD-1 antibody at 4℃ overnight. The membrane was washed with TBST and incubated with secondary antibody for 1 h at room temperature. Finally, the band was visualized by enhanced chemiluminescence (ECL).

For PD-L1 trap concentration analysis, the supernatant of EB was detected according to His Tag ELISA detection kit (GenScript).

**Photothermal effect**

In order to investigate the photothermal properties of EB@MPCM *in vitro*, different preparations (including MPM, MCM, MPCM and EB@MPCM) and different concentration of EB@MPCM were irradiated with 808 nm laser at 1.18 W/cm^2^. The temperature changes of different samples at different time were recorded by infrared thermal camera.

**EB activity analysis**

To examine the effect MPCM on EB activity, EB and EB@MPCM were diluted to OD_600_ of 0.2 in LB medium and incubated in a shaking incubator (37℃, 200 rpm). The OD_600_ values were recorded by a microplate reader at different time.

To examine the effect irradiation on EB activity, EB and EB@MPCM containing the same number of bacteria were irradiated with 808 nm laser at 1.18 W/cm^2^ for 3 min. The bacteria solution was coated on plate and the number of bacteria was counted.

**Hypoxic tendency of EB@MPCM**

The tendency of EB@MPCM to hypoxic environment was investigated by transwell migration experiment. To simulate hypoxia environment, glucose solution (0.4 mg/mL), glucose oxidase (0.5 KU), and catalase (0.5 KU) were added to the lower chamber of transwell. EGFP-labeled EB@MPCM was added to the upper chamber, and the number of bacteria in the upper and lower chamber was detected by flow cytometry and fluorescence microscopy at 15, 30 and 45 min, respectively.

**The shedding curve of EB@MPCM**

Rhodamine B-labeled MPM and MCM were dispersed in PBS and 1×10^8^ CFU engineered *E. coli* BL21 was added into the above solution. Then the solution was stirred at room temperature for 3 h. Rhodamine B-labeled EB@MPCM was dispersed in pH 6.8 PBS containing 100 μM H_2_O_2_. The bacterial precipitation was collected by centrifugation at different times (4℃, 5000 rpm, 10 min). The amount of MPM and MCM shed from EB was obtained by detecting the fluorescence of rhodamine B with fluorescence spectrophotometer

**The expression of cGAS-STING pathway-related protein**

4T1 cells were seeded in 6-well plates and incubated overnight. Cells were incubated with medium containing different concentrations of Mn^2+^ and different preparations (MNP, MNP+Laser, MnCl_2_, MPCM, MPCM+Laser) for 6 h, respectively. Laser is 808 nm laser irradiation at 1.18 W/cm^2^ for 3 min. The concentration of Mn^2+^ is 0.4 mM. Cells were collected by centrifugation at 1200 rpm for 5 min. 100 mL of cell lysate was added to the cells precipitate and the cells was lysed on ice for 40 min. Then, the protein supernatant was collected by centrifugation at 12000 rpm for 15 min at 4℃. Protein concentration was measured according to the BCA quantification kit method. The loading buffer was added to the protein sample and boiled at 100℃ for 10 min. Subsequently, the protein samples were separated by SDS-PAGE gels and the proteins on the gels were transferred to PVDF membranes. PVDF membrane containing protein was closed with 5%BSA at room temperature for 1 h and washed with TBST for 3 times. PVDF membrane was incubated with primary antibody overnight at 4℃ and washed with TBST for 3 times. Then the corresponding secondary antibodies were incubated at room temperature for 1 h, and the proteins were imaged with ECL luminescent solution after washing with TBST.

**The PD-L1 expression on 4T1 tumor cells surface**

4T1 cells were seeded in 6-well plates and incubated overnight. Medium containing MPCM, PD-L1 trap and MPCM+PD-L1 trap was added to the cells and cultured for 6 h. The concentration of Mn^2+^ and PD-L1 trap was 0.4 mM and 10 μg/mL, respectively. After centrifuging, 4T1 cells were added with BV650-PD-L1 antibody and incubated on ice for 30 min. the expression of PD-L1 on the surface of tumor cells was investigated by flow cytometry.

**Establishment of tumor-bearing mouse model and biodistribution of EB@MPCM**

4T1 tumor model was constructed in 6- to 8-week-old female BALB/c mice and B16-F10 tumor model was constructed in C57BL/6J mice. 4T1 cells and B16-F10 cells (2 × 10^6^) were resuspended in 100 μL of PBS and implanted in the axilla of mice. The tumor volume was monitored daily.

To investigate the distribution of EB *in vivo*, when the tumor volume reached ~100 mm^3^, EB@MPCM was intravenously injected into 4T1 tumor-bearing mice. The tumor, heart, liver, spleen, lung and kidney were extracted and homogenized at different times, respectively. The obtained liquid was coated on LB agar plates and cultured at 37℃. After 12 h, the colonies were counted and photographed.

For the distribution of EB@MPCM *in vivo*, when the tumor volume reached ~100 mm^3^, IR783, MPCM@IR783, EB@MNP@IR783 and EB@MPCM@IR783 were intravenously injected in to 4T1 tumor-bearing mice. Fluorescence images were captured at different time points after injection. After 72 h of administration, the mice were euthanized and major tissues were collected.

***In vivo* antitumor activity**

When the tumor volume was ~100 mm^3^, mice were separated randomly into ten groups and treated as follows: PBS, PBS+Laser, MPCM, MPCM+Laser, EB, EB+Laser, αPD-L1, EB@MPCM, EB@MPCM+αPD-L1, EB@MPCM+Laser (15 mg/kg of MPCM, 5 mg/kg of αPD-L1). The tumor sites were irradiated by 808 nm laser (1.18 W/cm^2^, 3 min). Tumor size was monitor daily and tumor volume was calculated by the formula: volume (mm^3^) = width^2^×length × 0.5. On day 10, major tissues including heart, liver, spleen, lung, kidney and tumor were collected and analyzed.

**The Mn content in tumor tissues of 4T1 tumor-bearing mice**

The 4T1 tumor-bearing mice was treated with MPCM and EB@MPCM at different time points. Then, the tumor tissues were collected and Mn content was detected by ICP-MS.

**Flow cytometry analysis of immune cells**

Immune cells change in tumor tissues by flow cytometry was based on previously reported methods.^[1]^ 0.3 g of 4T1 tumor tissues and B16-F10 tumor tissues was taken and cut into small pieces. 3 mL of tissues lysate was added and incubated in a shaking incubator (37℃, 150 rpm) for 40 min. An equal amount of termination solution was added and single-cell suspension was obtained by filtered. The cells suspension was stained with Zombie R718 Fixable Viability Stain (423115, Biolegend, 1:1000 dilution) on ice for 30 min. The single-cell suspension was divided into three parts and incubated with various antibodies. For panel 1, single-cell suspension was stained with Brilliant Violet 510™ anti-mouse CD45 (103137, Biolegend, clone number: 30-F11, 1:400 dilution), FITC anti-mouse CD3 (100203, Biolegend, clone number: 17A2, 1:500 dilution), APC anti-mouse CD4 (100411, Biolegend, clone number: GK1.5, 1:800 dilution), APC/Cyanine7 anti-mouse CD8 (100714, Biolegend, clone number: 53-6.7, 1:200 dilution), PE/Cyanine7 anti-mouse CD25 (101915, Biolegend, clone number: 3C7, 1:400 dilution), PE anti-mouse CD326 (118205, Biolegend, clone number: G8.8, 1:200 dilution), Brilliant Violet 650™ anti-mouse CD274 (124336, Biolegend, clone number: 10F.9G2, 1:400 dilution), Percp/Cyanine5.5 anti-mouse CD49b (103519, Biolegend, clone number: HMα2, 1:3000 dilution), Brilliant Violet 421™ anti-mouse Foxp3 (126419, Biolegend, clone number: MF-14, 1:400 dilution). For panel 2, single-cell suspension was stained with Brilliant Violet 510™ anti-mouse CD45 (103137, Biolegend, clone number: 30-F11, 1:400 dilution), PE anti-mouse CD86 (105008, Biolegend, clone number: GL-1, 1:200 dilution), Brilliant Violet 650™ anti-mouse CD80 (104732, Biolegend, clone number: 16-10A1, 1:400 dilution), Brilliant Violet 421™ anti-mouse CD11c (117329, Biolegend, clone number: N418, 1:800 dilution), PerCP/Cyanine5.5 anti-mouse CD11b (101227, Biolegend, clone number: M1/70, 1:800 dilution), APC/Cyanine7 anti-mouse Gr-1 (108424, Biolegend, clone number: RB6-8C5, 1:800 dilution). For panel 3, single-cell suspension was stained with Brilliant Violet 510™ anti-mouse CD45 (103137, Biolegend, clone number: 30-F11, 1:400 dilution), FITC anti-mouse CD3 (100203, Biolegend, clone number: 17A2, 1:500 dilution), APC/Cyanine7 anti-mouse CD8 (100714, Biolegend, clone number: 53-6.7, 1:200 dilution), Brilliant Violet 421™ anti-mouse PD-1 (109121, Biolegend, clone number: RMP1-30, 1:400 dilution), APC anti-mouse Tim3 (134007, Biolegend, clone number: B8.2412, 1:800 dilution), PE/Cyanine7 anti-mouse CD223 (LAG-3) (125225, Biolegend, clone number: C9B7W, 1:4000 dilution), PE anti-mouse IFN-γ (163503, Biolegend, clone number: W18272D, 1:800 dilution). For Brilliant Violet 421™ anti-mouse Foxp3 and PE anti-mouse IFN-γ, cells were permeabilized by True-Nuclear™ Transcription Factor Buffer Set (424401, Biolegend) according to the manufacturer’s protocols. Finally, the cells were washed with PBS and then detected by BD flow cytometer.

**ELISA assay**

Tumor tissues harvested from tumor-bearing mice was homogenized in tissue lysis working solution. The cell solution was lysed on ice for 40 min. the sample was centrifuged at 12000 rpm for 15 min. The protein supernatant was quantified by BCA kit and the contents of IFN-β, IL-6, TNF-α, IFN-γ were detected according to the ELISA kit instructions.

**Statistical analysis**

All statistical analyses were performed in GraphPad Prism 8.0. Data from the experiments were performed at least three times. The results were presented as means ± SD. Student’s t test was performed for statistical analysis. *P* value < 0.05 was considered statistically significant.


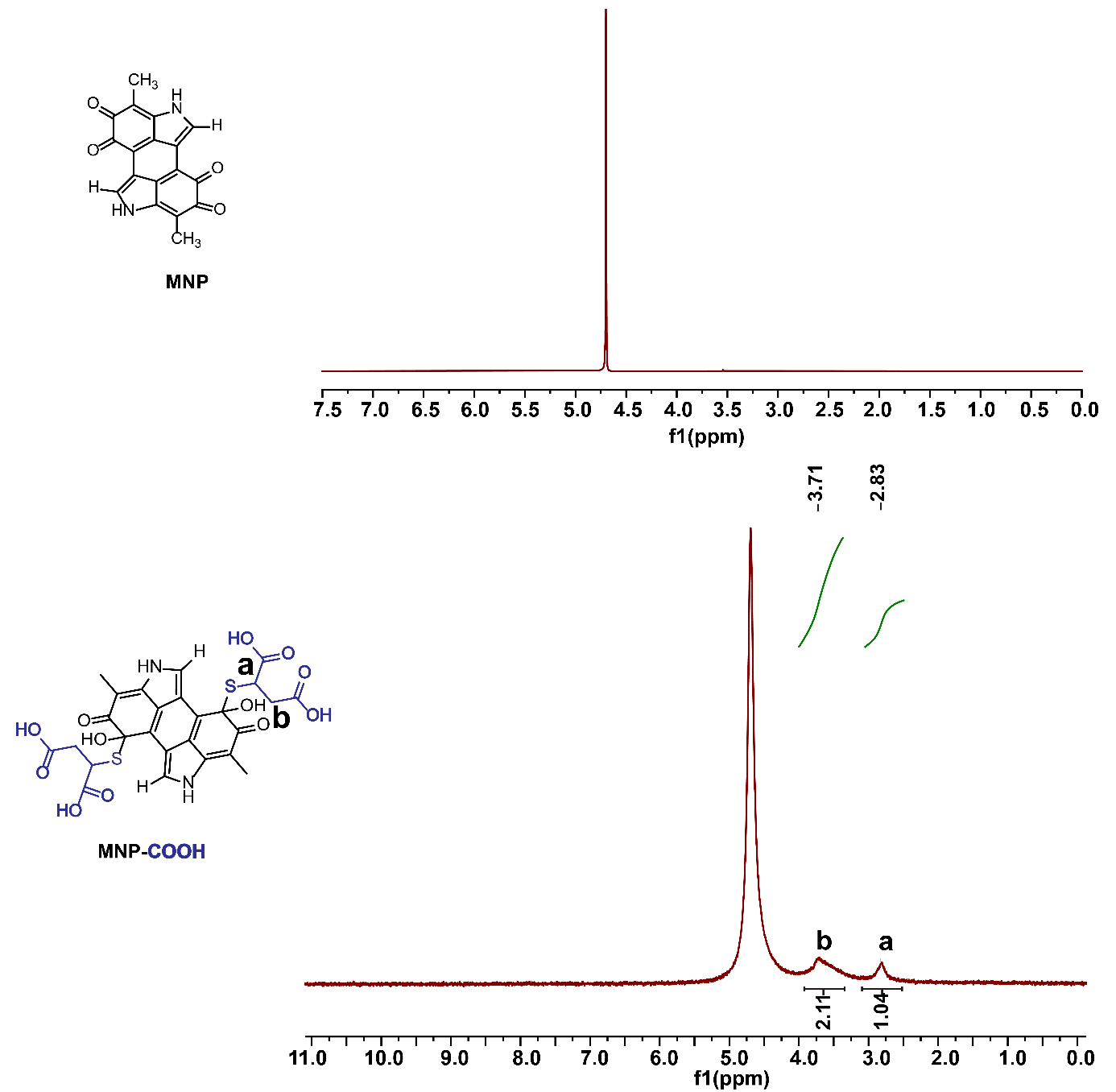
**Figure S1.** ^1^H NMR spectrum of MNP and MNP-COOH.


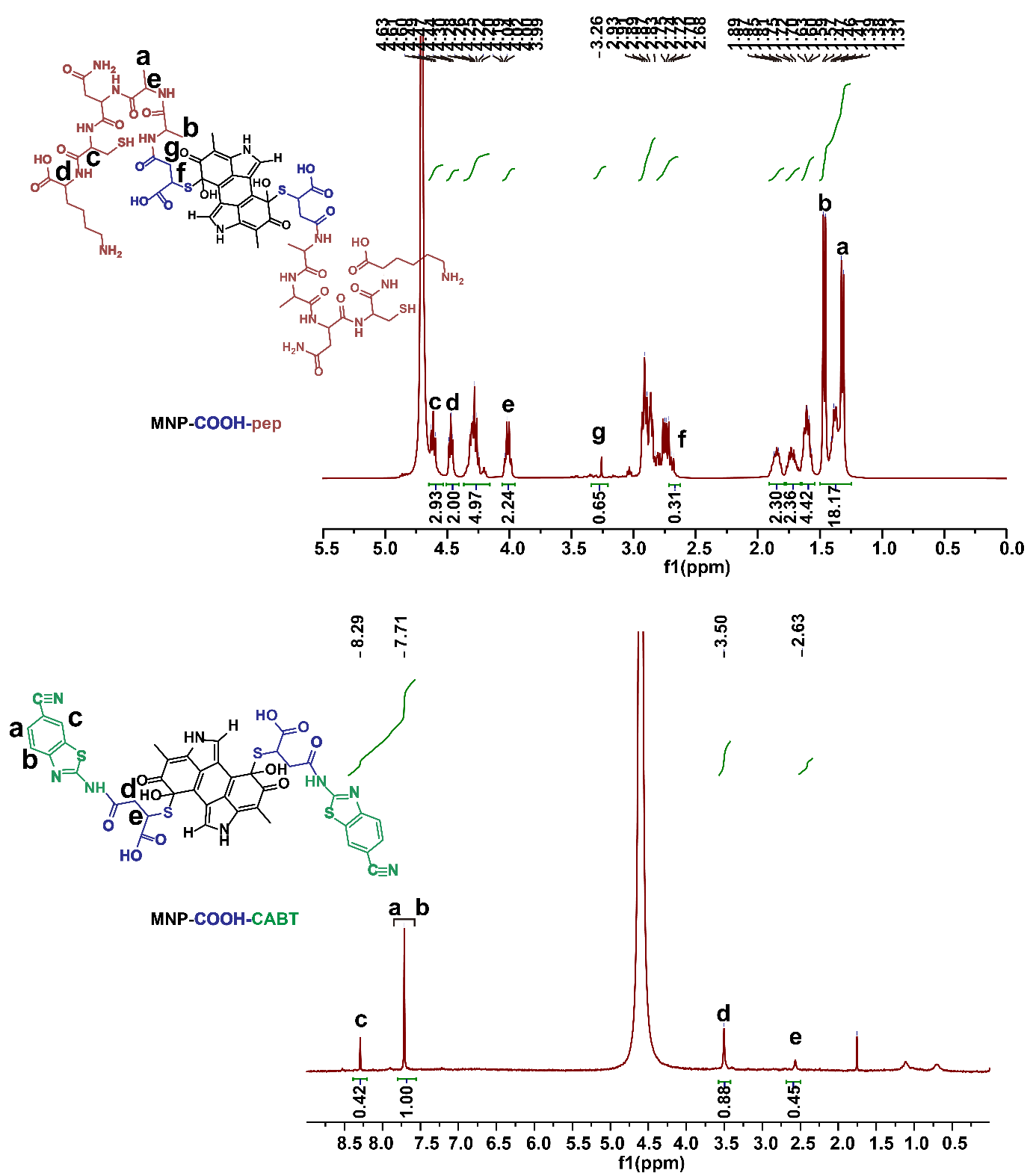
**Figure S2.** ^1^H NMR spectrum of MNP-pep and MNP-CABT.


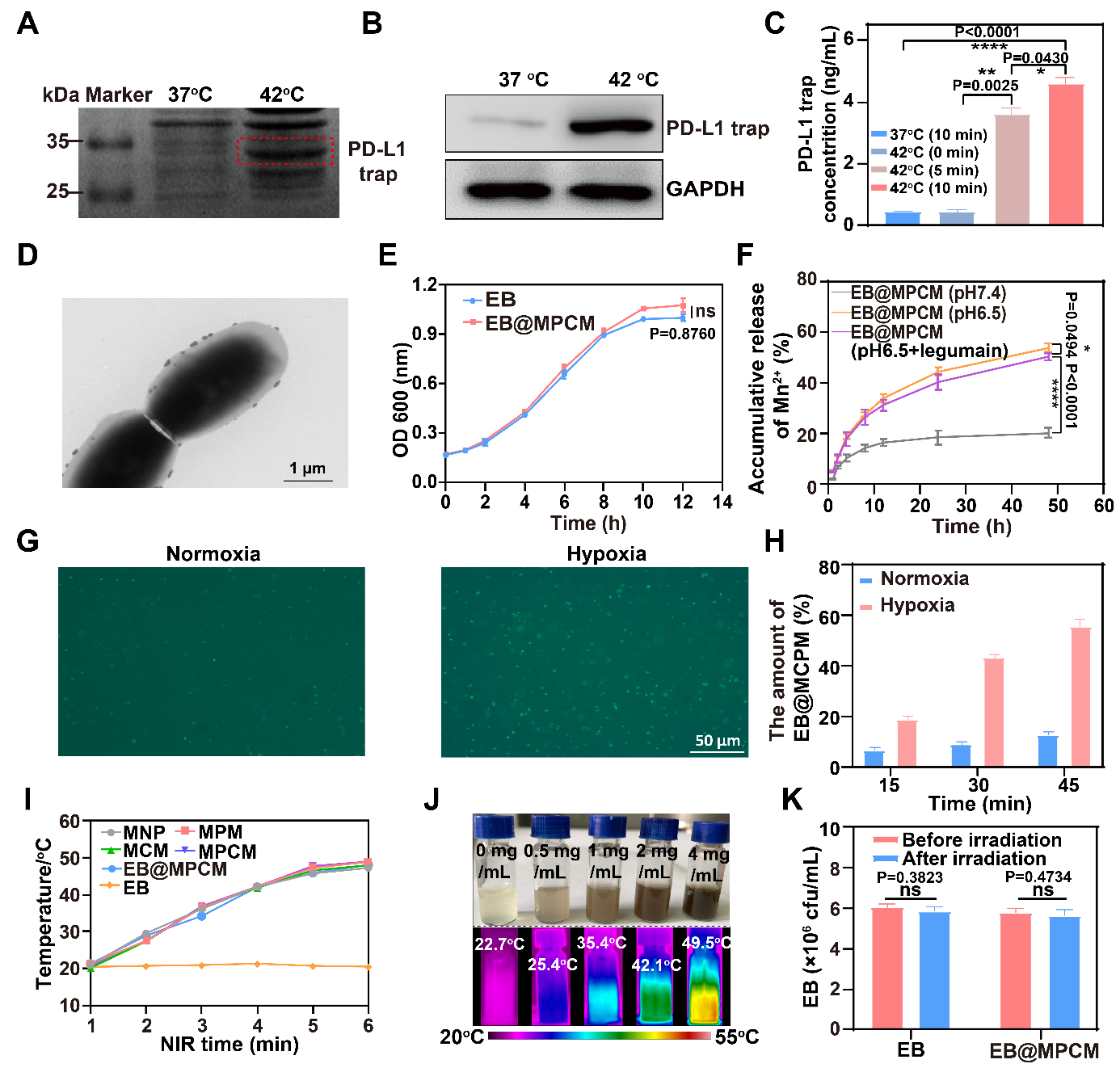


**Figure S3.** Thermosensitive PD-L1 trap secretion and hypoxic tropism of EB. (A-C) SDS-PAGE (A), western blot (B) detection of PD-L1 trap expression in EB after incubation at different temperatures. (C) ELISA kit detection of PD-L1 trap expression level after EB incubation at 37℃ and at 42℃ for different time. (D) The TEM image of EB@MPCM after incubation in pH 7.4 PBS for 12 h. (E) The growth curves of EB and EB@MPCM at different incubation time. (F) The release behavior of Mn^2+^ from EB@MPCM under different conditions. (G) Fluorescence pictures of EB@MPCM in normoxic and hypoxic environment after incubation for 45 min. (H) The amount of EB@MPCM in normoxic and hypoxic environment after incubation for different times. (I) Photothermal curves of different preparations under 808 nm laser irradiation. (J) Infrared thermal imaging of different concentration of EB@MPCM. (K) The concentration changes of EB in EB and EB@MPCM with or without laser irradiation.


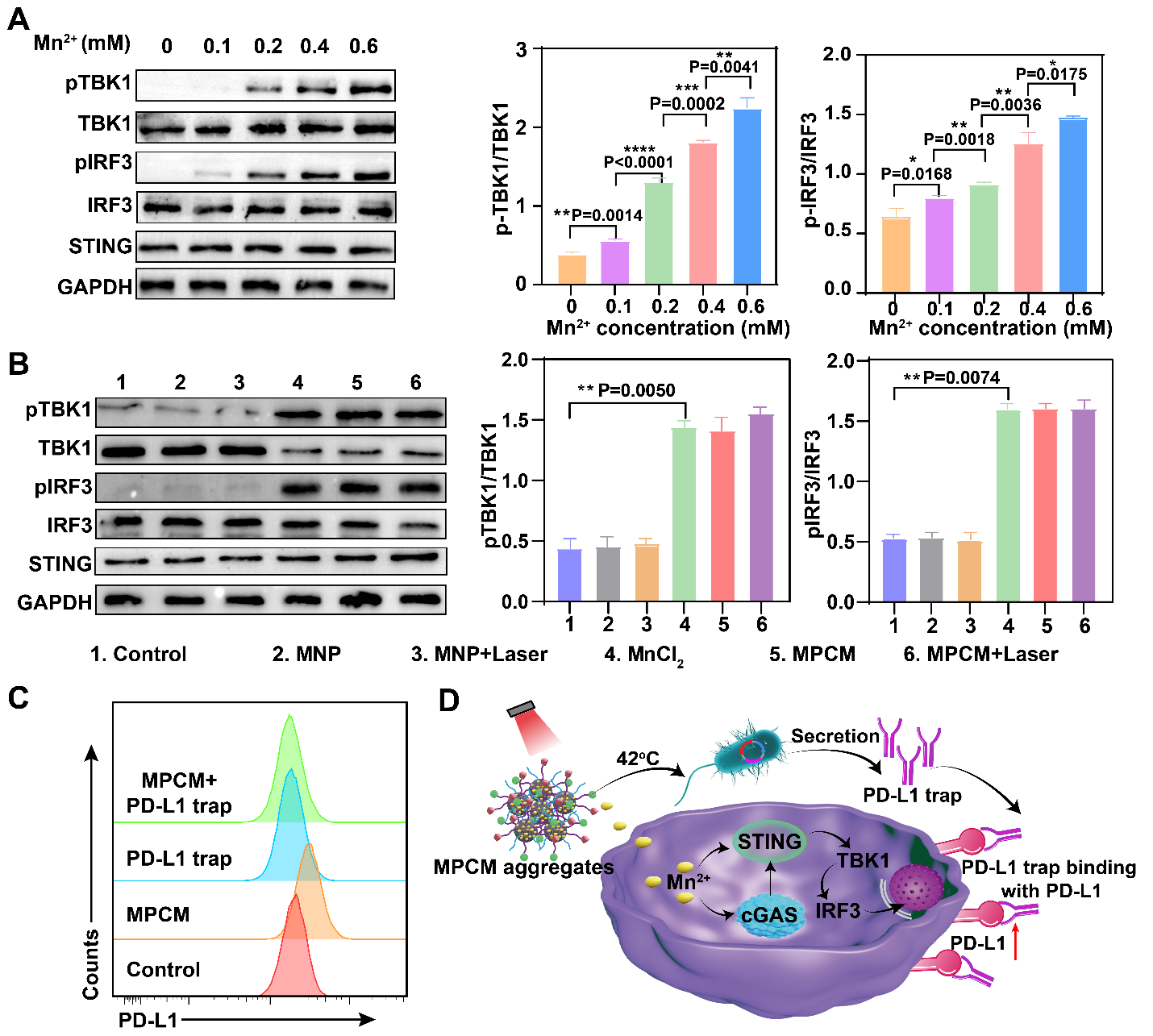


**Figure S4.** The expression of cGAS-STING pathway-related proteins and PD-L1 on tumor cells. (A, B) Western blot and semi-quantitative analysis of the expression of cGAS-STING pathway-related proteins in 4T1 tumor cells after incubation with different concentrations of Mn^2+^ (A) and different preparations (B). (C) Flow cytometry analysis of PD-L1 expression on 4T1 tumor cells after incubation with different preparations. (D) Schematic diagram of MPCM activating cGAS-STING signaling pathway and PD-L1 trap bound with PD-L1.


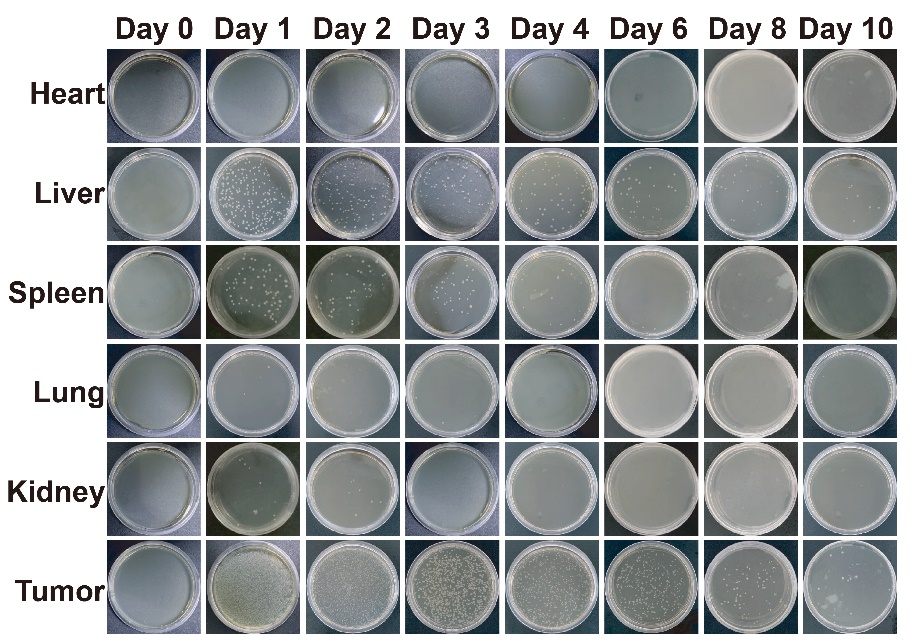


**Figure S5.** Distribution of EB@MPCM in different tissues at different times.


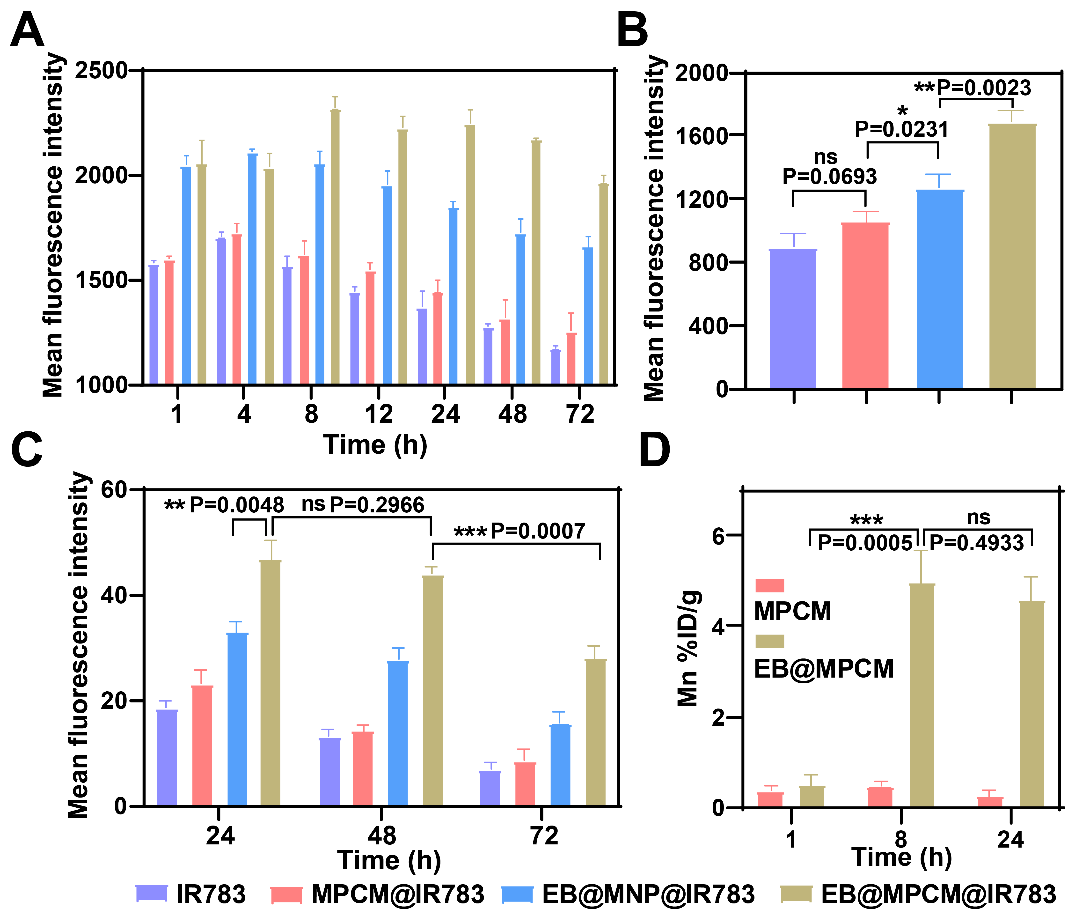


**Figure S6.** (A, B) Semi-quantitative analysis of IR783 fluorescence *in vivo* (A) and *ex vivo* (B) tumor tissues. (C) Semi-quantitative analysis of NR fluorescence in tumor tissues. (D) The Mn content in tumor tissues of 4T1 tumor-bearing mice treated with MPCM and EB@MPCM.


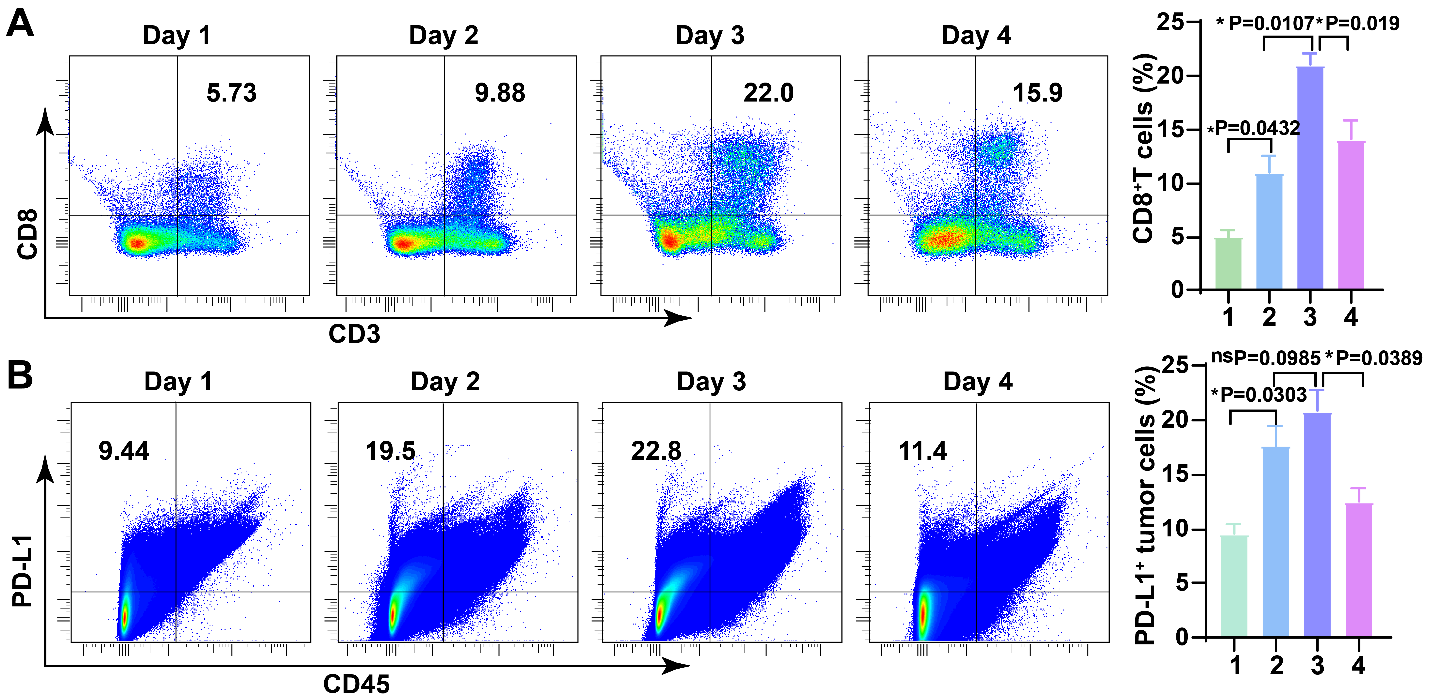


**Figure S7.** The expression levels of CD8^+^T and PD-L1^+^ tumor cells. (A, B) Flow cytometry and semi-quantitative analysis of the expression levels of CD8^+^T cells (A) and PD-L1^+^ tumor cells (B) in 4T1 tumor tissues.


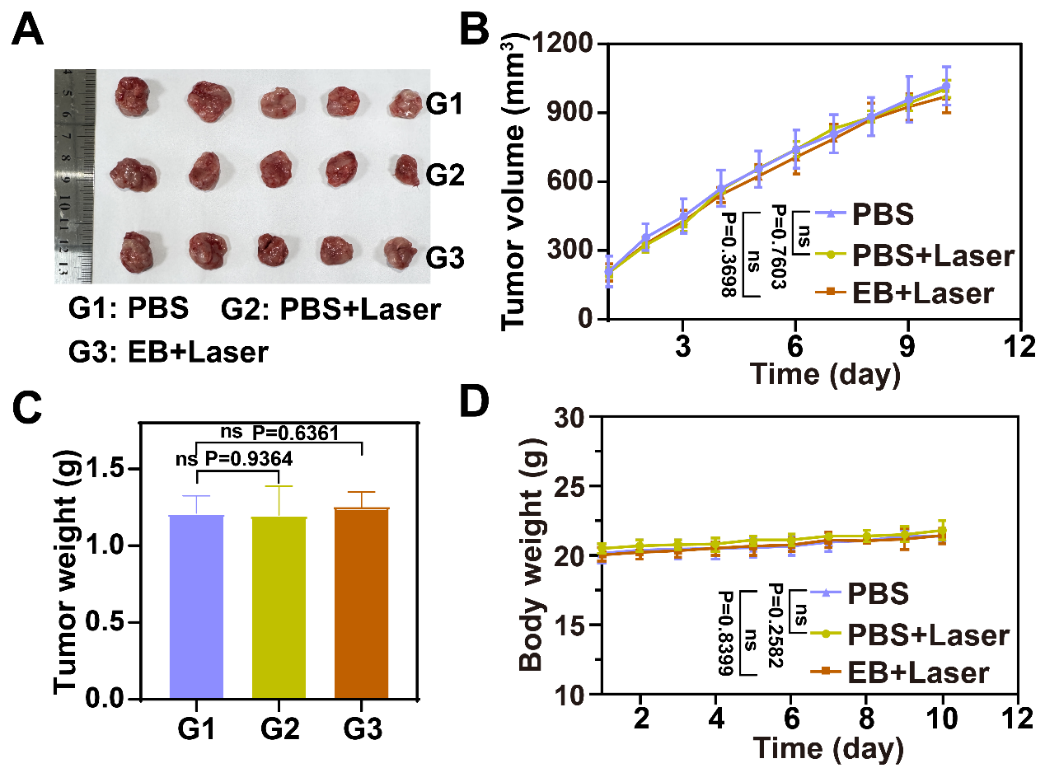


**Figure S8.** Antitumor efficacy of PBS+Laser and EB+Laser in 4T1 tumor-bearing mice. (A-D) *Ex vivo* tumor image (A), tumor growth curve (B), tumor weight (C) and body weight (D) of 4T1 tumor-bearing mice treated with different preparations.


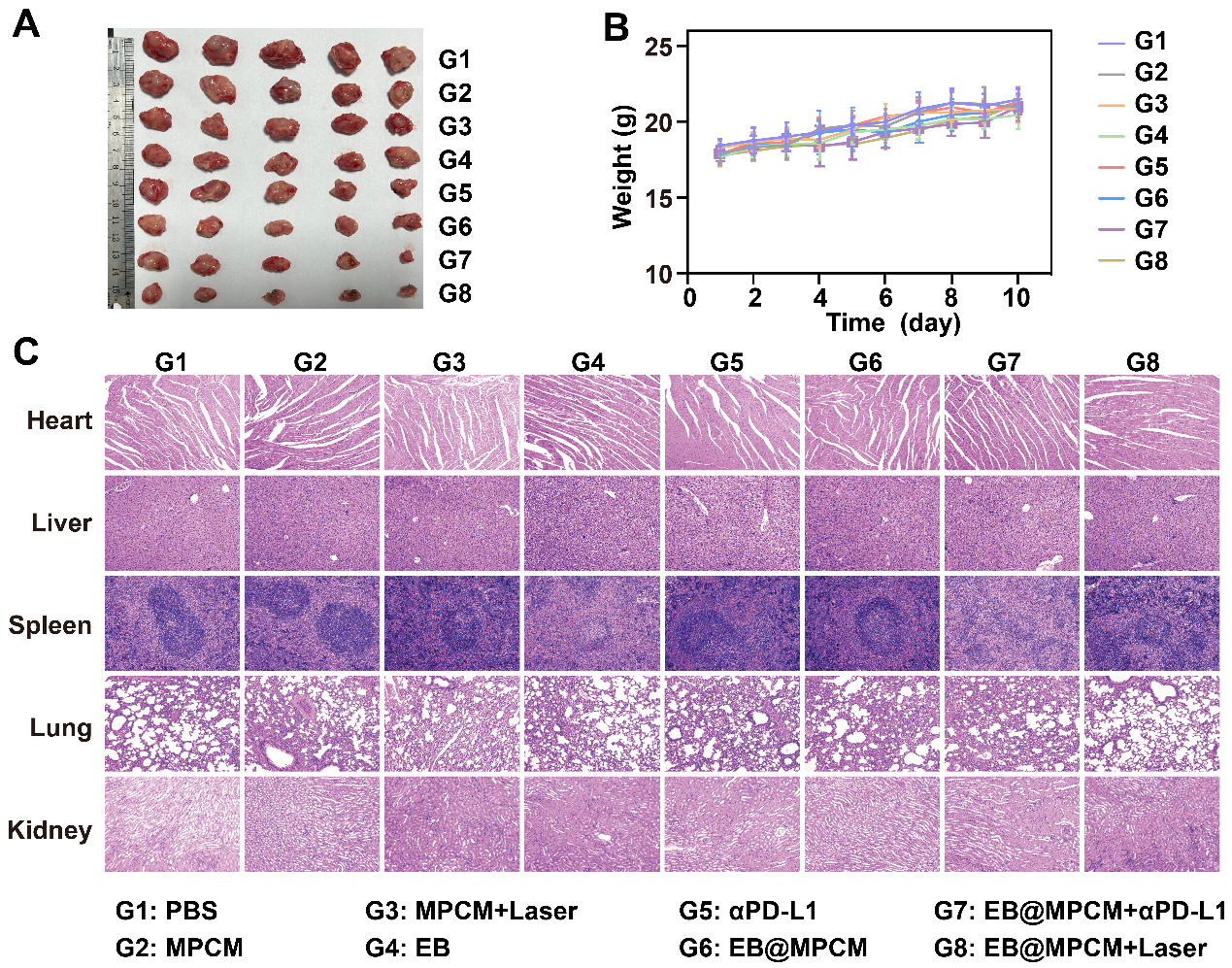


**Figure S9.** The tumor tissues images and biosafety of different preparations. (A, B) Tumor tissues images (A) and body weight (B) of 4T1 tumor-bearing mice treated with various formulations. (C) Representative images of H&E staining of different tissues.


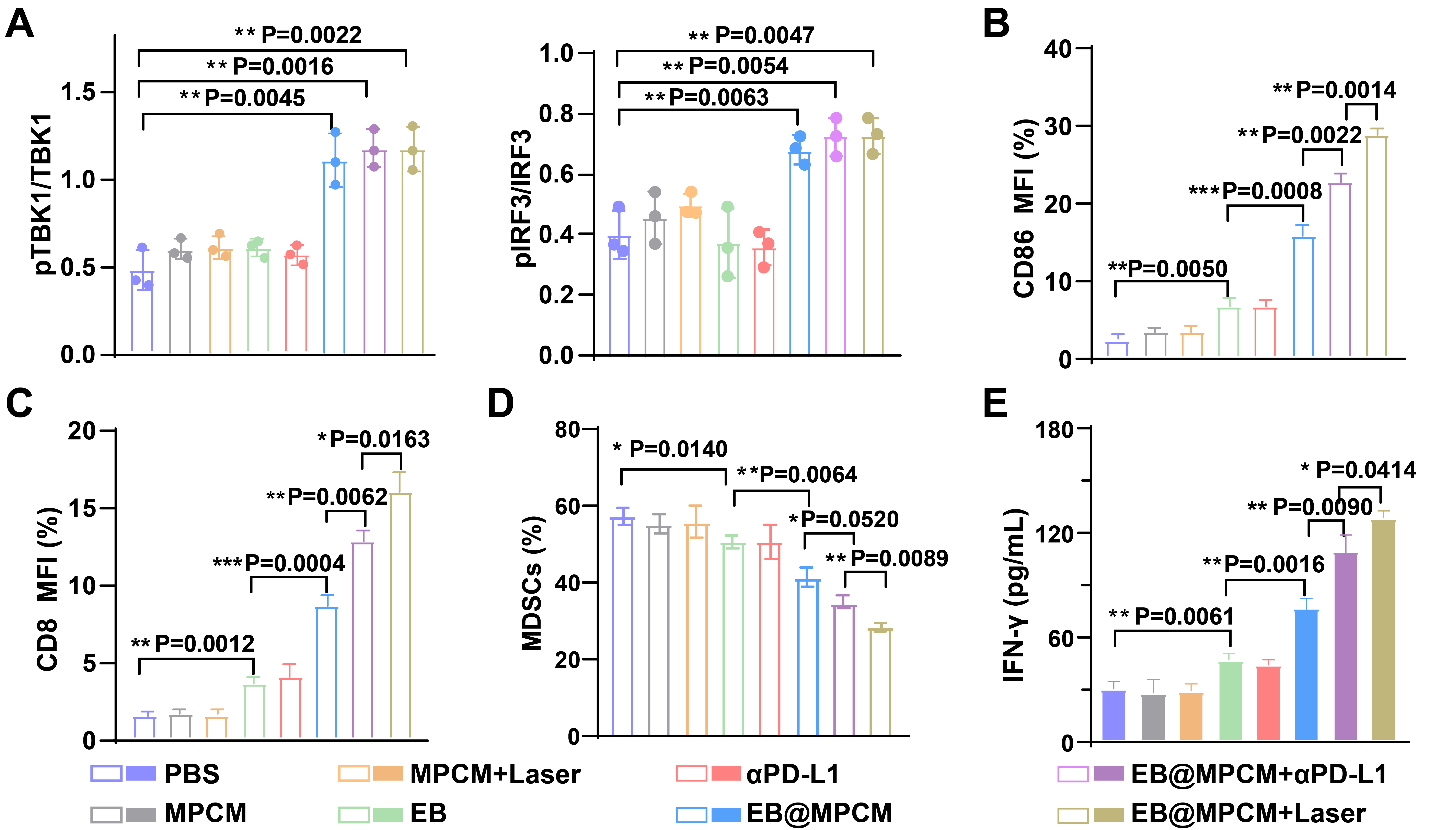


**Figure S10.** Semiquantitative analysis of cGAS-STING-related proteins and immune cells. (A) Semiquantitative analysis of pTBK1 and pIRF3 protein bands. (B, C) Mean fluorescence intensity of DCs (B) and CD8^+^T cells (C) in tumor slices. (D) Semiquantitative analysis of MDSCs in tumor tissues after treatment with different preparations. (E) Expression of IFN-γ in tumor tissues after treatment.


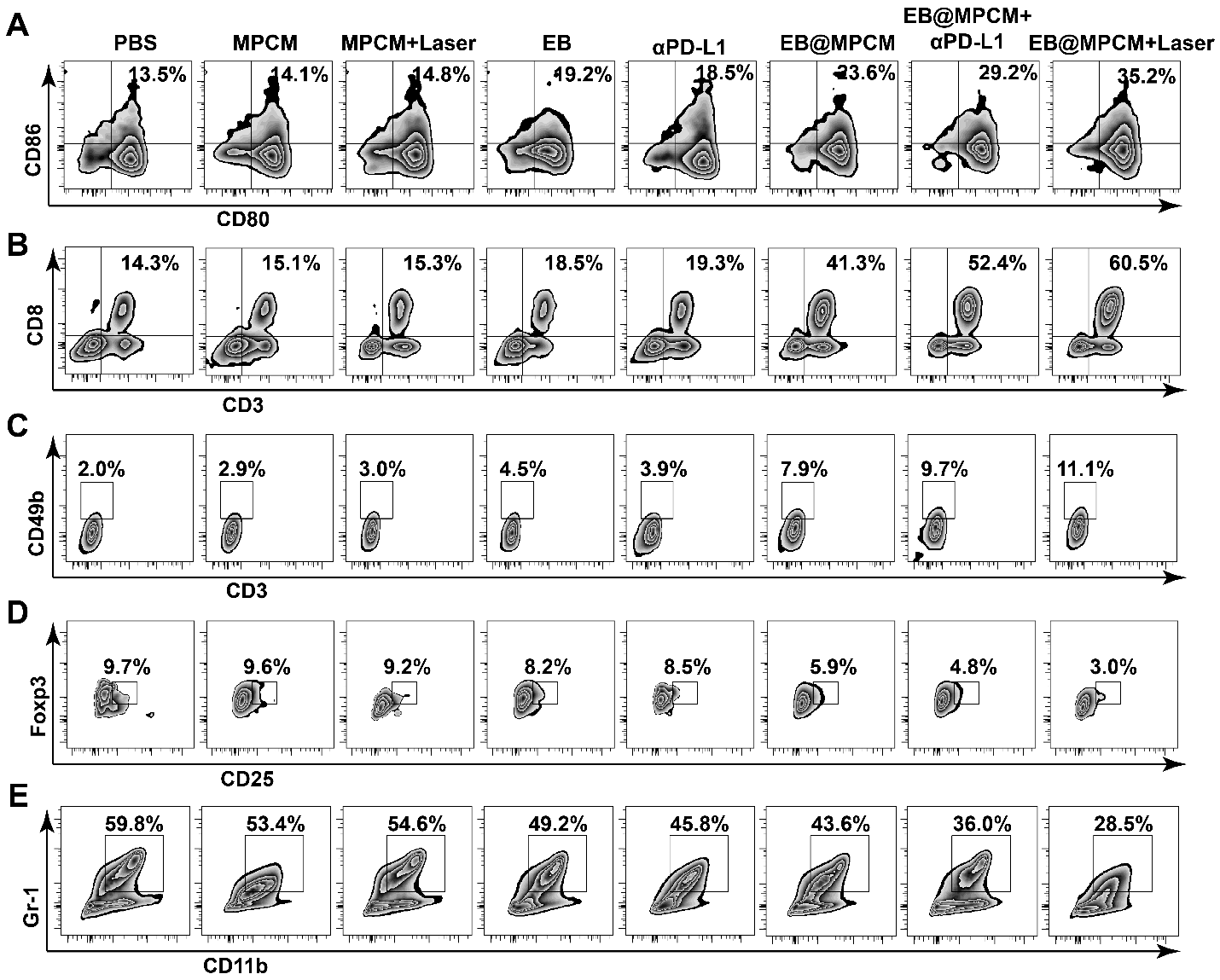


**Figure S11.** Immune cells analysis in tumor tissues of 4T1 tumor-bearing mice. (A-E) Flow cytometry analysis of DCs (CD80^+^CD86^+^) (A), CD8^+^T cells (CD3^+^CD8^+^) (B), NK cells (CD3^-^CD49b^+^) (C), Treg cells (CD25^+^Foxp3^+^) (D) and MDSCs (Gr-1^+^CD11b^+^) (E) in tumor tissues.


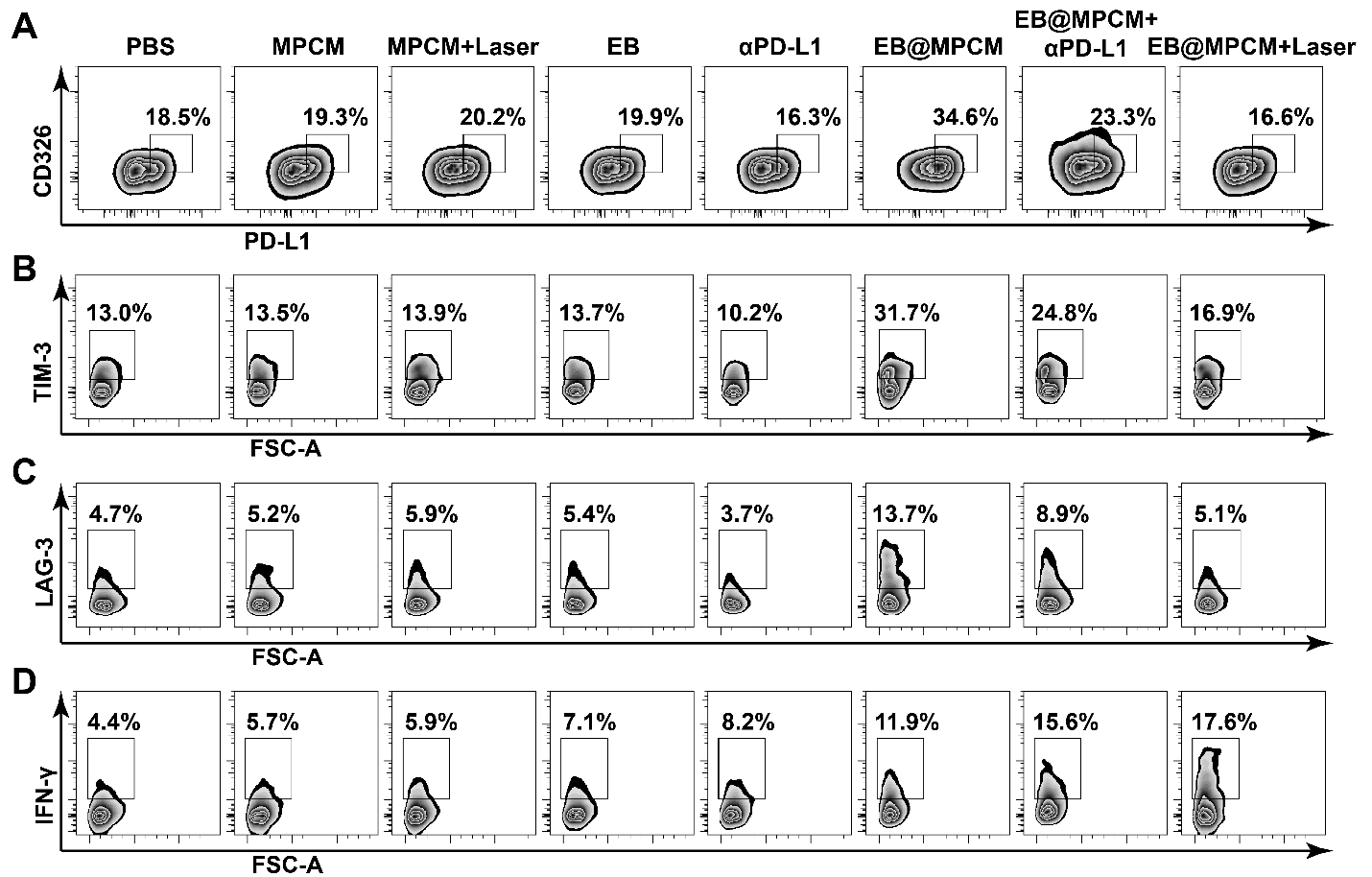


**Figure S12.** Expression of CD8^+^T cells surface molecules in tumor tissues of 4T1 tumor-bearing mice. (A-D) Flow cytometry analysis of PD-L1^+^ tumor cells (CD326^+^PD-L1^+^), TIM-3^+^, LAG-3^+^ and IFN-γ^+^ in CD8^+^T cells (CD3^+^CD8^+^) in tumor tissues.


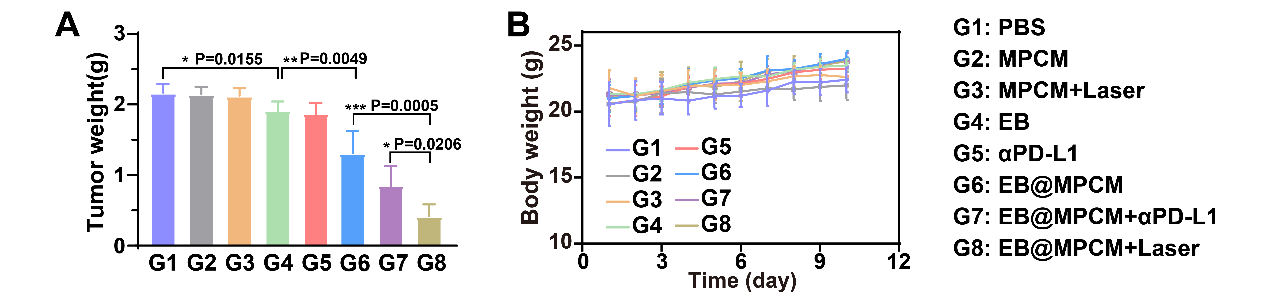


**Figure S13.** (A, B) Tumor weight (A) and body weight (B) of B16-F10 tumor-bearing mice treated with different preparations.


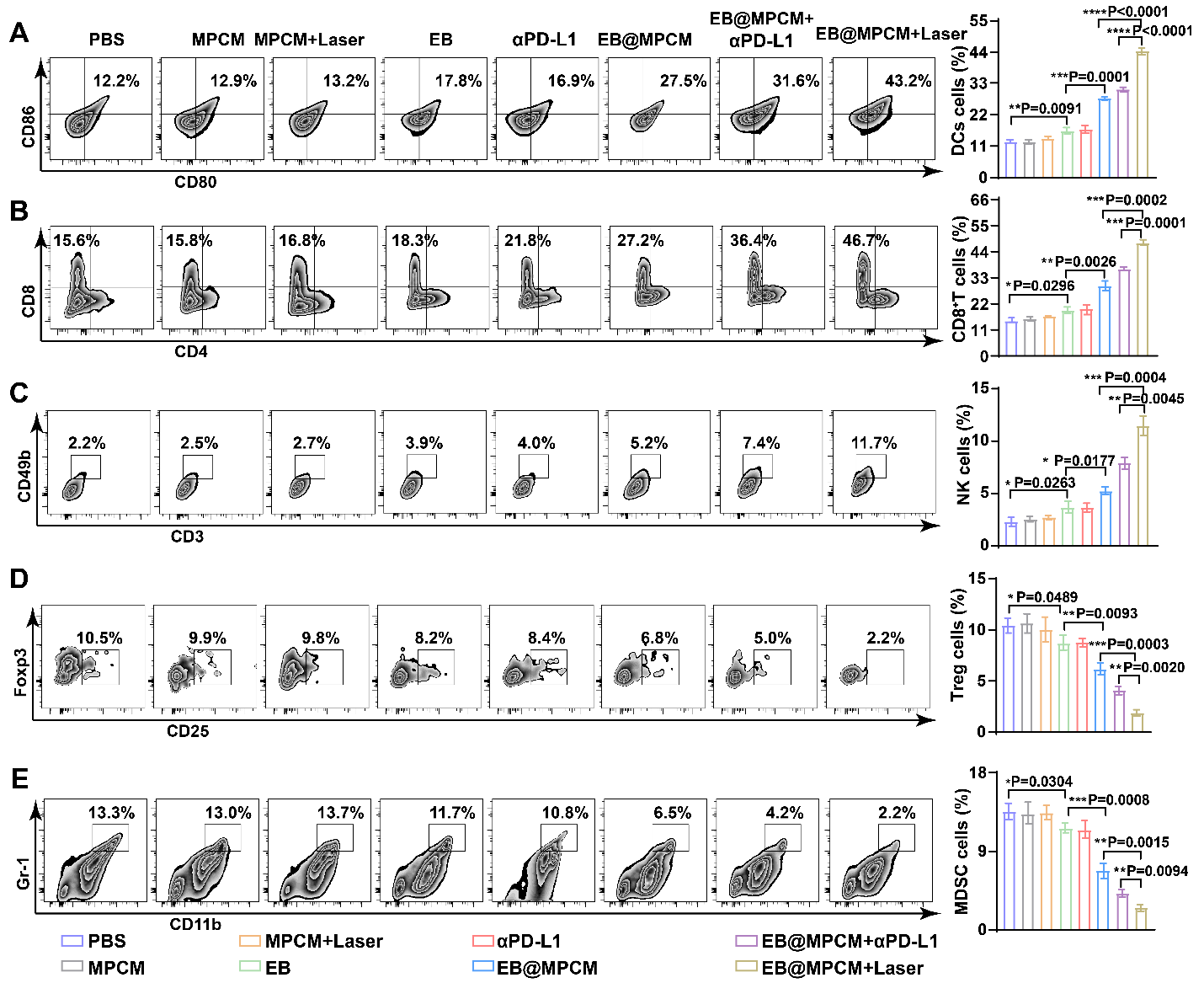


**Figure S14.** Immune cells analysis in tumor tissues of B16-F10 tumor-bearing mice. Flow cytometry and semiquantitative analysis of DCs (CD80^+^CD86^+^) (A), CD8^+^T cells (CD3^+^CD8^+^) (B), NK cells (CD3^-^CD49b^+^) (C), Treg cells (CD25^+^Foxp3^+^) (D) and MDSCs (Gr-1^+^CD11b^+^) (E) in tumor tissues of B16-F10 tumor-bearing mice.


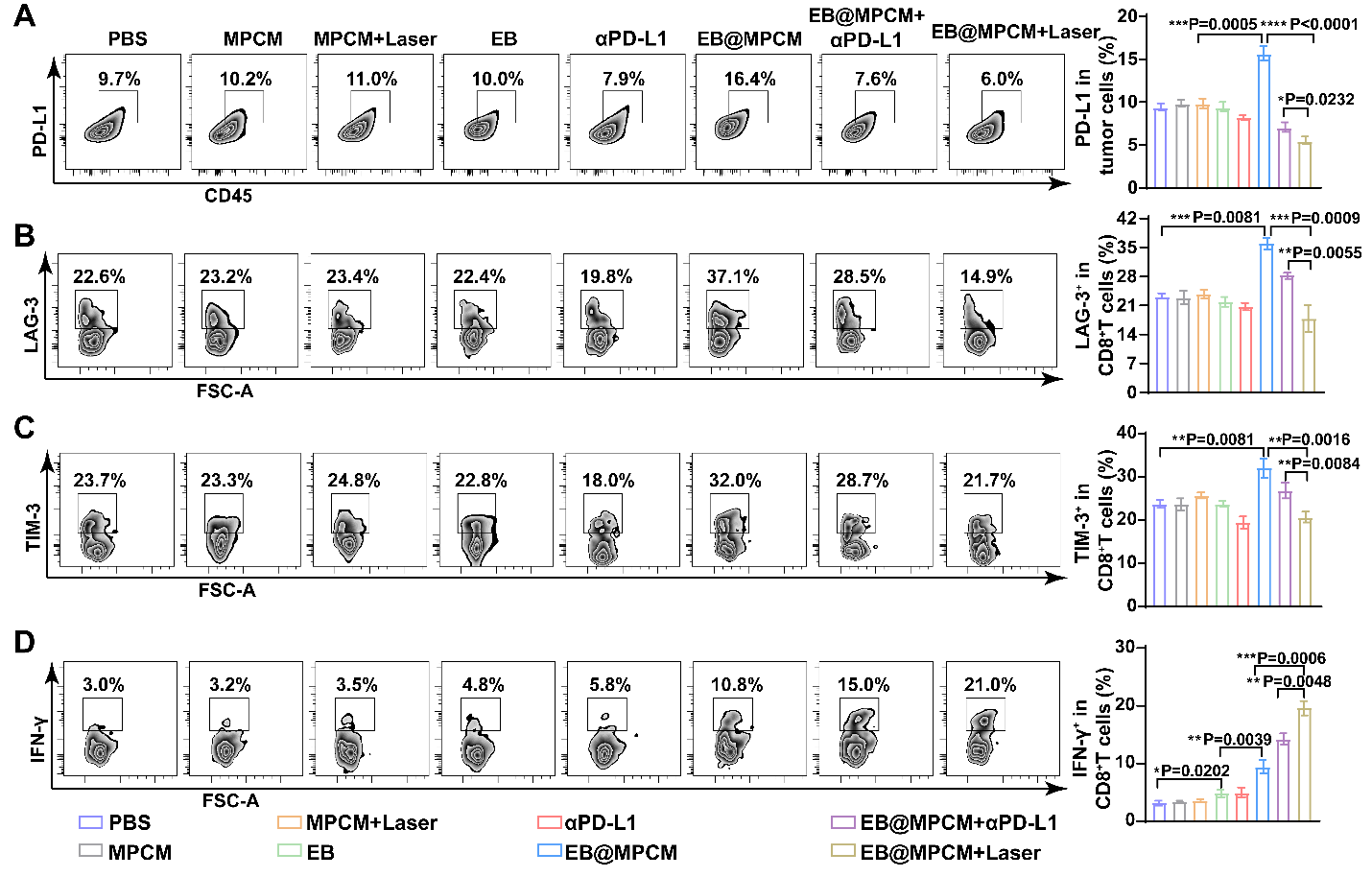


**Figure S15.** Expression of CD8^+^T cells surface molecules in tumor tissues of B16-F10 tumor-bearing mice. Flow cytometry and semiquantitative analysis of PD-L1^+^ tumor cells (CD45^-^PD-L1^+^), TIM-3^+^, LAG-3^+^ and IFN-γ^+^ in CD8^+^T cells (CD3^+^CD8^+^) in tumor tissues of B16-F10 tumor-bearing mice.

**Table S1.** Gene sequences of pelB and PD-L1 trap.

| Name | Sequence |
| --- | --- |
| pelB | ATGAAATACCTGCTGCCGACCGCTGCTGCTGGTCTGCTGCTCCTCGCTGCCCAGCCGGCGATGGCC |
| PD-L1 trap | ATGAAATGGGTTACCTTCATCTCTCTGCTGTTCCTGTTCTCTTCTGCTTACTCTGGTTCTTGGCTGCTGGAAGTTCCGAACGGTCCGTGGCGTTCTCTGACCTTCTACCCGGCTTGGCTGACCGTTTCTGAAGGTGCTAACGCTACCTTCACCTGCTCTCTGTCTAACTGGTCTGAAGACCTGATGCTGAACTGGAACCGTCTGTCTCCGTCTAACCAGACCGAAAAACAGGCTGCTTTCTGCAACGGTCTGTCTCAGCCGGTTCAGGACGCTCGTTTCCAGATCATCCAGCTGCCGAACCGTCACGACTTCCACATGAACATCCTGGACACCCGTCGTAACGACTCTGGTATCTACCTGTGCGGTGCTATCTCTCTGCACCCGAAAGCTAAAATCGAAGAATCTCCGGGTGCTGAACTGGTTGTTACCGAACGTATCCTGGAAGGTCCGCAGCCGCAGCCGAAACCGCAGCCGAAACCGGAACCGGAACCGCAGCCGCAGGGTGGTTCTGAAGAAGACCCGTGCGCTTGCGAATCTATCCTGAAATTCGAAGCTAAAGTTGAAGGTCTGCTGCAGGCTCTGACCCGTAAACTGGAAGCTGTTTCTGGTCGTCTGGCTGTTCTGGAAAACCGTATCATCGCTGCTGCTGGTGCTCCGGTTCCGTACCCGGACCCGCTGGAACCGCGTGGTGGTTCT |

References

[1] a)C. Liang, G. Zhang, L. Guo, X. Ding, H. Yang, H. Zhang, Z. Zhang, L. Hou, *Asian J. Pharm. Sci.* **2024**, 19, 100888; b) L. Hou, D. Chen, R. Wang, R. Wang, H. Zhang, Z. Zhang, Z. Nie, S. Lu, *angewandte* **2021**, 60, 6592.
